# Checklist of British and Irish Hymenoptera - Sawflies, ‘Symphyta’

**DOI:** 10.3897/BDJ.2.e1168

**Published:** 2014-08-29

**Authors:** Andrew D. Liston, Guy T. Knight, David A. Sheppard, Gavin R. Broad, Laurence Livermore

**Affiliations:** † Senckenberg Deutsches Entomologisches Institut, Müncheberg, Germany; ‡ Entomology, National Museums Liverpool, Liverpool, United Kingdom; § 32 South Street, Alford, Lincolnshire, United Kingdom; | The Natural History Museum, London, United Kingdom

## Introduction

The superfamilies Cephoidea, Orussoidea, Pamphilioidea, Siricoidea, Tenthredinoidea, Xiphydrioidea and Xyeloidea are combined as one checklist section, as the sawflies represent a distinctive assemblage of phytophagous (except Orussidae) Hymenoptera. More than fifty years have passed since the publication of the final part of Robert B. Benson’s three part identification key to the “Symphyta” of the British Isles (Benson 1935, Benson 1952, Benson 1958). Whilst the first two parts were based substantially on the acute monography by Eduard Enslin (Enslin 1912, Enslin 1913, Enslin 1914, Enslin 1915, Enslin 1916, Enslin 1917 and Enslin 1918), Benson’s treatment of the Nematinae represented an original and significant step forward in our knowledge of this subfamily. The results of subsequent research on the British and Irish sawfly fauna were collated by Quinlan (1978). Since then, renewed investigation of the taxonomy of West Palaearctic sawflies has led to a great number of taxonomic and nomenclatural changes, affecting many taxa occurring in the British Isles.

Of particular value in accessing the extensive but widely scattered literature on sawflies, is the online database “Electronic World Catalog of Symphyta (ECatSym)” (Blank et al. 2012). Unfortunately, the only identification guide to the European “Symphyta” published since 1958 with a more than local geographical scope, by Zhelochovtsev (1988), suffers from weaknesses, such as the reliance placed on the opinions and illustrations of E. Lindqvist in its treatment of the Nematinae. Mistakes in translation from Russian make the later English language edition of this work even more difficult to use.

Special problems are attached to the interpretation of records for some species not found in the British Isles since their inclusion in the works of Leach (1817) and Stephens (1835). Some of these are probably extinct, but their presence in the British Isles has in many cases never been unequivocally proved: see for example Benson (1943). We therefore maintain the practice, as in Fitton et al. (1978), of marking names of such taxa with a preceding “?”. Further, the taxonomic status of some of the nominal taxa described from the British Isles by Hill, Stephens, Newman and Cameron is still unclear, particularly where no type material has been located. The number of confirmed British sawfly species now stands at 537, compared to 471 in the 1978 checklist (Quinlan 1978; Broad 2014). Note that, since the publication of the introduction section (Broad 2014) a further family, Heptamelidae, has been recognised (Malm and Nyman 2014), including two British species previously included in Tenthredinidae.

Authorship and date of publication of original descriptions by various authors follow mainly the bibliographic research of Taeger and Blank (1996), Taeger and Blank (2006), Blank and Taeger (1998), Blank et al. (2009) and Taeger et al. (2010).

The classification of genera used here is largely based on the system of Benson (1951-1958). This has the advantages of being relatively simple and widely known. Detailed phylogenetic studies on several major lineages of Tenthredinidae are still lacking. However, some definite conclusions on generic groupings were reached by Nyman et al. (2006) on the Nematinae and Leppänen et al. (2012) on the Fenusini of the Blennocampinae. For the higher Nematinae, the full taxonomic and nomenclatural consequences of the recently proposed phylogeny have not yet been drawn. Far fewer genera will in future be recognised. For the present, we revert here to a generic classification that is similar to that used by Benson (1958), even though we are aware that *Pachynematus*, for example, is not monophyletic.

Genus and species group names are included selectively in the synonymy. For a complete current synonymy of all these, including important misidentifications, see ECatSym (Blank et al. 2012). A name is only included as a synonym when it fulfils one or both of the following criteria:

- has at some time been used for a taxon occurring in the British Isles;
- is used in widely consulted works on taxonomy or distribution, or in original accounts of biological characters / descriptions of immature stages.

The general scope and rationale for the checklist are covered by Broad (2014). As for the rest of the checklist, the Channel Islands fauna is excluded: see Sheppard (1990) on the sawfly fauna of the Channel Islands. There is a lack of data on the sawflies of the Isle of Man. It should be noted that several species were mistakenly listed as occurring in Scotland by Liston (1995).

Some of the diversity of British sawflies is illustrated in Figs 1, 2, 3, 4.

## Checklists

### Superfamily CEPHOIDEA Newman, 1834

#### Cephidae

Newman, 1834

#### Cephinae

Newman, 1834

#### Cephini

Newman, 1834

#### Calameuta

Konow, 1896

MONOPLOPUS Konow, 1896
HAPLOCEPHUS Benson, 1935

#### Calameuta filiformis

(Eversmann, 1847)

Cephus filiformis Eversmann, 1847
Cephus elongatus (Vollenhoven, 1858, *Cephus*)
Cephus arundinis (Giraud, 1863, *Cephus*)
Cephus analis (Klug, 1803): Stephens, 1835 misident.

##### Distribution

England, Wales

#### Calameuta pallipes

(Klug, 1803)

Astatus pallipes Klug, 1803
Cephus pusilla (Stephens, 1835, *Cephus*)
Cephus immaculata (Stephens, 1835, *Cephus*)
Cephus cultrarius (Hartig, 1837, *Cephus*)
Cephus pallidipes (Dalla Torre, 1894, *Cephus*)
Cephus phthisica (Fabricius, 1805, *Cephus*): misspelling

##### Distribution

England, Scotland, Wales, Ireland

#### Cephus

Latreille, 1802

#### Cephus nigrinus

Thomson, 1871

Cephus pallipes (Klug, 1803): Stephens, 1835 misident.

##### Distribution

England

#### Cephus pygmeus

(Linnaeus, 1767)

Sirex pygmeus Linnaeus, 1767
Astatus floralis (Klug, 1803, *Astatus*)
Cephus atripes Stephens, 1835
Cephus pygmaeus : misspelling

##### Distribution

England, Wales

#### Cephus spinipes

(Panzer, 1800)

Banchus spinipes Panzer, 1800
Cephus pilosulus Thomson, 1871
Cephus pusillus : Stephens, 1835 misident.
Cephus cultratus : Eversmann, 1847 misident. and misspelling of *Cephus cultrarius* Hartig

##### Distribution

England, Wales

#### Trachelus

Jurine, 1807

CEPHA Billberg, 1820
ATEUCHOPUS Konow, 1896
EUMETABOLUS Schulz, 1906
TRACHELASTATUS Morice & Durrant, 1915
NEATEUCHOPUS Benson, 1935
MICROCEPHUS Benson, 1935

#### Trachelus tabidus

(Fabricius, 1775)

Sirex tabidus Fabricius, 1775
Sirex macilentus (Fabricius, 1793, *Sirex*)
Cephus mandibularis (Serville, 1823, *Cephus*)
Cephus nigritus (Serville, 1823, *Cephus*)

##### Distribution

England, Wales

#### Trachelus troglodyta

(Fabricius, 1787)

Sirex troglodyta Fabricius, 1787
Trachelus niger (Harris, 1779): Cameron, 1890 misident.

##### Distribution

✝England

##### Notes

Extinct in Britain

#### Hartigiini

Enslin, 1914

#### Janus

Stephens, 1829

EPHIPPIONOTUS Costa, 1860

#### Janus cynosbati

(Linnaeus, 1758)

Tenthredo cynosbati Linnaeus, 1758
Cephus femoratus (Curtis, 1830, *Cephus*)
Cephus niger (Brischke, 1892, *Cephus*)

##### Distribution

England

##### Notes

Nomenclature follows Blank et al. (2009).

#### Janus luteipes

(Lepeletier, 1823)

Cephus luteipes Lepeletier, 1823
Janus connectens Stephens, 1829
Janus bifrons Newman, 1838

##### Distribution

England, Wales

#### Phylloecus

Newman, 1838

HARTIGIA Schiødte, 1839
MACROCEPHUS Schlechtendal, 1878
COPIOSOMA W. F. Kirby, 1882
ADIRUS Konow, 1899

##### Notes

Synonymy of *Hartigia* under *Phylloecus* is discussed by Liston and Prous (2014).

#### Phylloecus faunus

Newman, 1838

Cephus helleri (Taschenberg, 1871, *Cephus*)
Cephus albomaculatus (J. Stein, 1876, *Cephus*)

##### Distribution

England

##### Notes

Added by Liston and Prous (2014) on the basis of the lectotype in the collections of Oxford University Museum of Natural History that Newman (1838) stated was collected “in the vicinity of London”.

#### Phylloecus linearis

(Schrank, 1781)

Tenthredo linearis Schrank, 1781
Cephus quinquefasciata (Stephens, 1835, *Cephus*)
Cephus major (Eversmann, 1847, *Cephus*)
Macrocephus agrimoniae (Goury, 1912, *Macrocephus*)

##### Distribution

England, Wales

#### Phylloecus niger

(M. Harris, 1779)

Sirex niger  M. Harris, 1779
Astatus satyrus (Panzer, 1801, *Astatus*)
Cephus brachyptera (Damianitsch, 1866, *Cephus*)

##### Distribution

England, Wales

#### Phylloecus xanthostoma

(Eversmann, 1847)

Cephus xanthostoma Eversmann, 1847
Macrocephus ulmariae (Schlechtendal, 1878, *Macrocephus*)

##### Distribution

England, Scotland, Wales

### Superfamily ORUSSOIDEA Newman, 1834

#### Orussidae

Newman, 1834

#### Orussus

Latreille, 1797

ORYSSUS Fabricius, 1798

#### Orussus abietinus

(Scopoli, 1763)

Sphex abietina  Scopoli, 1763

##### Distribution

?England

##### Notes

Recorded from Kent and Devon by Stephens (1835), but see comments in Benson (1951).

### Superfamily PAMPHILIOIDEA Cameron, 1890

#### Megalodontesidae

Konow, 1897

Megalodontidae preocc.

#### Megalodontes

Latreille, 1802

TARPA Fabricius, 1804

##### Notes

If any member of this genus was ever present in England, then only *Megalodontes cephalotes* seems at all likely: according to Leach (1817) and Stephens (1835), found near Bristol and Plymouth. See Taeger (2002) for current taxonomy. of the genus.

Species of *Megalodontes* removed from the British and Irish list:

[***Megalodontes panzeri*** (Leach, 1817), ***Megalodontes plagiocephalus*** (Fabricius, 1804)] Up to three separate *Megalodontes* species have at times been considered to have been present in England. Neither *Megalodontes panzeri* nor *Megalodontes plagiocephalus*, on the basis of their currently known distributions and habitat requirements, probably ever occurred in the British Isles.

#### Megalodontes cephalotes

(Fabricius, 1781)

Tenthredo cephalotes Fabricius, 1781
Tarpa klugii (Leach, 1817, *Tarpa*)
Tarpa spissicornis (Klug, 1824, *Tarpa*)
Megalodontes klugi : misspelling

##### Distribution

?England

#### Pamphiliidae

Cameron, 1890

##### Notes

Unplaced species of Pamphiliidae, treated as nomen oblitum by Blank et al. (2009):

***Tenthredo variegata*** Hill, 1773

#### Cephalciinae

Benson, 1945

#### Acantholyda

Costa, 1894

ITYCORSIA Konow, 1897

#### Acantholyda erythrocephala

(Linnaeus, 1758)

Tenthredo erythrocephala Linnaeus, 1758

##### Distribution

England, Scotland

#### Acantholyda posticalis

(Matsumura, 1912)

Lyda posticalis Matsumura, 1912
Tenthredo stellata (Christ, 1791, *Tenthredo*) preocc.
Acantholyda ?pinivora Enslin, 1918
Acantholyda nemoralis (Linnaeus, 1758): Thomson, 1871 misident.

##### Distribution

England, Scotland

#### Cephalcia

Panzer, 1803

CEPHALCIA Jurine, 1801 suppressed
CEPHALEIA Jurine, 1807

##### Notes

Species of *Cephalcia* removed from the British and Irish list:

[***Cephalcia arvensis*** Panzer, 1803] Recorded by Shinohara (2002) but deleted by Shaw (2003) because material probably not of British origin.

#### Cephalcia lariciphila

(Wachtl, 1898)

Cephaleia lariciphila Wachtl, 1898
Cephalcia alpina (Klug, 1808): misident.
Cephalcia falleni (Dalman, 1823): misident.
Cephalcia annulata (Hartig, 1837): misident.

##### Distribution

England, Scotland, Wales

#### Pamphiliinae

Cameron, 1890

#### Neurotoma

Konow, 1897

GONGYLOCORSIA Konow, 1897
GONGYLOCORISA : Ashmead, 1898 misspelling

#### Neurotoma mandibularis

(Zaddach, 1866)

Lyda mandibularis Zaddach, 1866

##### Distribution

England

#### Neurotoma saltuum

(Linnaeus, 1758)

Tenthredo saltuum Linnaeus, 1758
Tenthredo flaviventris (Retzius, 1783, *Tenthredo*)
Lyda fasciata (Curtis, 1831, *Lyda*)

##### Distribution

England, Wales

#### Pamphilius

Latreille, 1802

LYDA Fabricius, 1804
ANOPLOLYDA Costa, 1894
BACTROCERUS Konow, 1897

##### Notes

Species of *Pamphilius* removed from the British and Irish list:

[***Lyda jucundus*** (Eversmann, 1847, *Lyda*) (*Pamphilius nemorum* auctt.)] See Liston et al. (2006)

#### Pamphilius albopictus

(Thomson, 1871)

Lyda albopicta  Thomson, 1871

##### Distribution

Scotland

##### Notes

Added by Shinohara (1998)

#### Pamphilius balteatus

(Fallén, 1808)

Lyda balteata  Fallén, 1808
Pamphilius cingulatus Latreille, 1812

##### Distribution

England, Scotland, Wales, Ireland

#### Pamphilius betulae

(Linnaeus, 1758)

Tenthredo betulae Linnaeus, 1758

##### Distribution

England

#### Pamphilius fumipennis

(Curtis, 1831)

Lyda fumipennis Curtis, 1831

##### Distribution

England, Wales

#### Pamphilius gyllenhali

(Dahlbom, 1835)

Pamphilius gyllenhali See ICZN (2005)  Opinion 2101.
Lyda gyllenhali Dahlbom, 1835

##### Distribution

England, Scotland, Wales, Ireland

#### Pamphilius histrio

Latreille, 1812

Pamphilius flaviventris (Retzius, 1783): Stephens, 1835 misident.

##### Distribution

England, Scotland, Wales

#### Pamphilius hortorum

(Klug, 1808)

Lyda hortorum Klug, 1808
Pamphilius hortorum ssp. bicinctus Benson, 1945

##### Distribution

England, Scotland, Wales, Ireland

#### Pamphilius inanitus

(Villers, 1789)

Tenthredo inanita  Villers, 1789

##### Distribution

England, Scotland, Wales, Ireland

#### Pamphilius latifrons

(Fallén, 1808)

Pamphilius latifrons See ICZN (2005), Opinion 2101.
Lyda latifrons Fallén, 1808
Lyda maculosus (Zaddach, 1866, *Lyda*)

##### Distribution

England

#### Pamphilius pallipes

(Zetterstedt, 1838)

Lyda pallipes Zetterstedt, 1838

##### Distribution

England, Scotland, Wales, Ireland

#### Pamphilius stramineipes

(Hartig, 1837)

Lyda stramineipes Hartig, 1837
Pamphilius arbustorum (Fabricius, 1793): Cameron, 1885 misident.

##### Distribution

Scotland

#### Pamphilius sylvarum

(Stephens, 1835)

Lyda sylvarum Stephens, 1835
Lyda nigricornis (Vollenhoven, 1858, *Lyda*)

##### Distribution

England, Wales

#### Pamphilius sylvaticus

(Linnaeus, 1758)

Tenthredo sylvatica  Linnaeus, 1758

##### Distribution

England, Scotland, Wales, Ireland

#### Pamphilius vafer

(Linnaeus, 1767)

Tenthredo vafra  Linnaeus, 1767
Tenthredo depressus (Schrank, 1781, *Tenthredo*)

##### Distribution

England, Scotland, Wales, Ireland

#### Pamphilius varius

(Serville, 1823)

Lyda varia  Serville, 1823
Pamphilius vafer (Linnaeus, 1767): misident.

##### Distribution

England, Scotland, Wales, Ireland

### Superfamily SIRICOIDEA Billberg, 1820

#### Siricidae

Billberg, 1820

#### Sirex

Linnaeus, 1761

#### Sirex areolatus

(Cresson, 1867)

Urocerus areolatus  Cresson, 1867

##### Distribution

#England

##### Notes

Introduced, not established.

#### Sirex juvencus

(Linnaeus, 1758)

Ichneumon juvencus  Linnaeus, 1758
Sirex nigricornis Acerbi, 1802
Sirex dubia W. F. Kirby, 1882

##### Distribution

England, Scotland, Wales

##### Notes

*Sirex atricornis* is considered to be a valid species (Schiff et al. 2012), which may occur in Scotland (Viitasaari and Midtgaard 1989).

#### Sirex noctilio

Fabricius, 1793

Sirex melanocerus Thomson, 1871

##### Distribution

England, Scotland, Wales, Ireland

#### Sirex torvus

M. Harris, 1779

Sirex duplex Shuckard, 1837
Sirex cyaneus Fabricius, 1781: misident.

##### Distribution

England, Scotland, Wales, Ireland

##### Notes

A species of Central European origin, thought to be attached mainly to *Abies* spp. and not conspecific with the Nearctic *Sirex cyaneus* (Schiff et al. 2012).

#### Tremex

Jurine, 1807

XYLOTERUS Geoffroy, 1762
XYLOECEMATIUM Heyden, 1868

#### Tremex columba

(Linnaeus, 1763)

Sirex columba  Linnaeus, 1763

##### Distribution

#England, #Scotland

##### Notes

Introduced, not established.

#### Urocerus

Geoffroy, 1785

UROCERUS Geoffroy, 1762
XANTHOSIREX Semenov-Tian-Shanskij, 1921

#### Urocerus albicornis

(Fabricius, 1781)

Sirex albicornis  Fabricius, 1781
Sirex stephensii (W. F. Kirby, 1882, *Sirex*)

##### Notes

#Introduced, not established.

#### Urocerus augur

(Klug, 1803)

Sirex augur  Klug, 1803
Sirex bimaculata (Donovan, 1808, *Sirex*)
Sirex cedrorum (Smith, 1860, *Sirex*)
Urocerus fantoma (Fabricius, 1781)

##### Distribution

England, Scotland, Ireland

##### Notes

Established in Ireland. *Urocerus augur*, *Urocerus fantoma* and *Urocerus tardigradus* have at times been recorded as introductions to the British Isles, but only *Urocerus augur* is regarded as established (O'Connor et al. 1997). All were treated as valid species by Smith (1978), although *Urocerus tardigradus* had for a long time been placed as a synonym of *Urocerus fantoma*. Benson 1958 (ii, supplement to section a) added *Urocerus tardigradus* to the British list with “*Urocerus fantoma* Fabricius auctt. nec Fabricius” as its synonym and separated this in a key from “*Urocerus fantoma fantoma* (= *Urocerus augur augur* Klug)”. The reasons for this treatment are not clear. No type specimens are known to exist for either *Urocerus fantoma* or *Urocerus tardigradus*, but Fabricius (1781) in his short original description of *Sirex fantoma* describes the legs as yellow, whereas *Urocerus augur* has extensively black tibiae. European specimens previously identified as *Urocerus tardigradus* have so far all proved to belong to *Urocerus fantoma* (A. Taeger, pers. comm.). We revert here to treating *Urocerus tardigradus* as a synonym of *Urocerus fantoma*.

#### Urocerus californicus

Norton, 1869

##### Notes

#Introduced, not established.

#### Urocerus fantoma

(Fabricius, 1781)

Sirex fantoma  Fabricius, 1781
Sirex tardigradus (Cederhjelm, 1798, *Sirex*)

##### Notes

#Introduced, not established.

#### Urocerus flavicornis

(Fabricius, 1781)

Sphex flavicornis  Fabricius, 1781
Sirex bizonatus (Stephens, 1835, *Sirex*)

##### Distribution

#England

##### Notes

*Sirex bizonatus*, collected 'near London', is a synonym of the Nearctic *Urocerus flavicornis* (Schiff et al. 2012). Presumably an introduction, not known to be established in the British Isles.

#### Urocerus gigas

(Linnaeus, 1758)

Ichneumon gigas  Linnaeus, 1758
Sirex bizonatus (Stephens, 1835, *Sirex*)
Urocerus taiganus Benson, 1943

##### Distribution

England, Scotland, Wales, Ireland

#### Xeris

Costa, 1894

#### Xeris spectrum

(Linnaeus, 1758)

Ichneumon spectrum  Linnaeus, 1758

##### Distribution

England, Wales, Ireland

### Superfamily TENTHREDINOIDEA Latreille, 1803

#### Argidae

Konow, 1890

#### Arginae

Konow, 1890

#### Arge

Schrank, 1802

CRYPTUS Jurine, 1801 suppressed
HYLOTOMA Latreille, 1802

#### Arge berberidis

Schrank, 1802

##### Distribution

England, Wales

##### Notes

Added by Halstead (2004).

#### Arge ciliaris

(Linnaeus, 1767)

Tenthredo ciliaris Linnaeus, 1767
Hylotoma coerulea (Klug, 1814, *Hylotoma*)
Hylotoma corusca (Zaddach, 1859, *Hylotoma*)

##### Distribution

England, Scotland, Ireland

#### Arge cyanocrocea

(Forster, 1771)

Tenthredo cyanocrocea  Forster, 1771
Tenthredo coerulescens (Fabricius, 1775, *Tenthredo*)
Hylotoma coerulea (Latreille, 1805, *Hylotoma*)

##### Distribution

England, Scotland, Wales, Ireland

#### Arge enodis

(Linnaeus, 1767)

Tenthredo enodis  Linnaeus, 1767
Tenthredo coeruleipennis (Retzius, 1783, *Tenthredo*)

##### Distribution

England

#### Arge expansa

(Klug, 1834)

Hylotoma expansa  Klug, 1834
Arge clavicornis (Fabricius, 1781): misident.

##### Distribution

Scotland, Ireland

#### Arge fuscipes

(Fallén, 1808)

Hylotoma fuscipes Fallén, 1808
Arge clavicornis (Fabricius, 1781): misident.
Hylotoma violacea (Klug, 1814, *Hylotoma*)
Hylotoma atrocoerulea (Serville, 1823, *Hylotoma*)
Arge fuscinervis Lindqvist, 1974

##### Distribution

England, Scotland, Wales, Ireland

#### Arge gracilicornis

(Klug, 1814)

Hylotoma gracilicornis Klug, 1814
Tenthredo coerulescens (Geoffroy, 1785, *Tenthredo*) preocc.
Tenthredo incolorata (Christ, 1791, *Tenthredo*) nom. ob.
Hylotoma pilicornis (Leach, 1817, *Hylotoma*)
Hylotoma cyanella (Klug, 1834, *Hylotoma*)
Hylotoma crassa (Konow, 1884, *Hylotoma*)

##### Distribution

England, Scotland, Wales, Ireland

#### Arge melanochra

(Gmelin, 1790)

Tenthredo melanochra Gmelin, 1790
Hylotoma femoralis (Klug, 1814, *Hylotoma*)
Hylotoma dimidiata (Serville, 1823, *Hylotoma*) preocc.
Hylotoma nigritarsis (Klug, 1834, *Hylotoma*)
Hylotoma bicolor (Gimmerthal, 1846, *Hylotoma*)
Arge fuliginata Konow, 1907
Arge melanochroa : misspelling

##### Distribution

England

#### Arge metallica

(Klug, 1834)

Hylotoma metallica  Klug, 1834

##### Distribution

Ireland

#### Arge nigripes

(Retzius, 1783)

Tenthredo nigripes Retzius, 1783
Arge enodis (Linnaeus, 1767): misident.
Hylotoma anglica (Leach, 1817, *Hylotoma*)

##### Distribution

England, Scotland, Wales

#### Arge ochropus

(Gmelin, 1790)

Tenthredo ochropus Gmelin, 1790
Arge rosincola Schrank, 1802
Hylotoma rosarum (Klug, 1814, *Hylotoma*)
Arge rosae (Linnaeus, 1758): misident.
Arge ochropa : misspelling

##### Distribution

England

#### Arge pagana

(Panzer, 1797)

Tenthredo pagana Panzer, 1797
Hylotoma ?stephensii (Leach, 1817, *Hylotoma*)

##### Distribution

England, Wales

##### Notes

*Arge stephensii* differs very markedly, in the extreme development of pale body markings, from all known W. Palaearctic populations of *Arge pagana*. Traditionally, *Arge stephensii* has been regarded as an endemic British subspecies of *Arge pagana*. Further studies [here a DNA analysis might offer good clues] are definitely required. The possibility cannot be excluded that *Arge stephensii* is a separate species originally introduced from the E. Palaearctic or Oriental Regions, where *Arge* is both extremely species-rich and poorly investigated.

#### Arge rustica

(Linnaeus, 1758)

Tenthredo rustica Linnaeus, 1758
Tenthredo atrata (Forster, 1771, *Tenthredo*)
Cryptus segmentaria (Panzer, 1803, *Cryptus*)
Hylotoma klugii (Leach, 1817, *Hylotoma*)
Hylotoma leachii (Stephens, 1835, *Hylotoma*)

##### Distribution

England

#### Arge ustulata

(Linnaeus, 1758)

Tenthredo ustulata Linnaeus, 1758

##### Distribution

England, Scotland, Wales, Ireland

#### Sterictiphorinae

Rohwer, 1911

#### Aprosthema

Konow, 1899

COPIDOCEROS Forsius, 1921

#### Aprosthema fusicorne

(Thomson, 1871)

Aprosthema fusicorne  *Schizocera fusicornis* Thomson, 1871

##### Distribution

England

##### Notes

Added by Vikberg (2004).

#### Aprosthema melanurum

(Klug, 1814)

Schizocera friesei (Konow, 1895, *Schizocera*)
Schizocera alfkeni (Konow, 1895, *Schizocera*)
Aprosthema melanura : misspelling

##### Distribution

England

#### Aprosthema tardum

(Klug, 1814)

Hylotoma tarda  Klug, 1814

##### Distribution

England

##### Notes

Added by Vikberg (2004).

#### Sterictiphora

Billberg, 1820

SCHIZOCERUS Berthold, 1827
SCHIZOCERA Lepeletier & Serville, 1828
CYPHONA Dahlbom, 1835
SCHIZOCEROS Konow, 1899
STERICTOPHORA Benson, 1951: misspelling

#### Sterictiphora angelicae

(Panzer, 1799)

Cryptus villersii (Leach, 1817, *Cryptus*)
Sterictiphora melanocephala (Fabricius, 1798): misident.
Sterictiphora furcata (Villers, 1789): misident.

##### Distribution

England, Wales

#### Sterictiphora geminata

(Gmelin, 1790)

Tenthredo geminata  Gmelin, 1790
Cryptus pallipes (Leach, 1817, *Cryptus*)

##### Distribution

England, Scotland, Wales, Ireland

#### Blasticotomidae

Thomson, 1871

#### Blasticotoma

Klug, 1834

#### Blasticotoma filiceti

Klug, 1834

##### Distribution

England, Wales, Ireland

#### Cimbicidae

Curtis, 1825

#### Abiinae

Benson, 1951

##### Notes

Benson (1951) treated *Abia* and *Zaraea* as belonging to two different tribes. The differences between these certainly do not warrant this (Lorenz and Kraus 1957, Taeger 1998).

#### Abia

Leach, 1817

ZARAEA Leach, 1817
AENOABIA Kangas, 1946
AUROABIA Kangas, 1946

#### Abia aenea

(Klug, 1820)

Abia bifida (Thomson, 1871, *Abia*)
Abia bigens (Kangas, 1946, *Abia*)
Abia lonicerae (Linnaeus, 1758): Enslin, 1917 misident.

##### Distribution

England

#### Abia candens

Konow, 1887

##### Distribution

England, Scotland, Wales, Ireland

#### Abia fasciata

(Linnaeus, 1758)

Tenthredo fasciata  Linnaeus, 1758

##### Distribution

England, Scotland, Wales, Ireland

#### Abia lonicerae

(Linnaeus, 1758)

Abia nigricornis Leach, 1817

##### Distribution

England, Wales, Ireland

#### Abia sericea

(Linnaeus, 1767)

Tenthredo sericea  Linnaeus, 1767

##### Distribution

England, Scotland, Wales, Ireland

#### Cimbicinae

Curtis, 1825

#### Cimbicini

Curtis, 1825

#### Cimbex

Olivier, 1790

CRABRO Geoffroy, 1762 suppressed
CLAVELLARIUS Olivier, 1789 suppressed
CLAVELLARIA Lamarck, 1801
PALAEOCIMBEX Semenov Tian-Shanskij, 1935
ALLOCIMBEX Zirngiebl, 1953

#### Cimbex connatus

(Schrank, 1776)

Tenthredo connata Schrank, 1776

##### Distribution

England, Wales, Ireland

#### Cimbex femoratus

(Linnaeus, 1758)

Tenthredo femorata Linnaeus, 1758
Cimbex europaea Leach, 1817
Tenthredo sylvarum (Fabricius, 1793, *Tenthredo*)
Cimbex varians Leach, 1817

##### Distribution

England, Scotland, Wales, Ireland

#### Cimbex luteus

(Linnaeus, 1758)

Tenthredo lutea Linnaeus, 1758
Cimbex griffinii Leach, 1817
Cimbex annulata (Geoffroy, 1785): Leach, 1817 misident.

##### Distribution

England, Scotland, Wales

#### Cimbex quadrimaculatus

(Müller, 1766)

Tenthredo quadrimaculata  Müller, 1766
Crabro humeralis (Geoffroy, 1785, *Crabro*)
Tenthredo axillaris (Panzer, 1801, *Tenthredo*)

##### Distribution

✝England

##### Notes

Extinct in Britain; presence in British Isles based on one female (NHM) said to have been collected near Salisbury (Leach 1817, Stephens 1835).

#### Trichiosomini

Benson, 1951

#### Pseudoclavellaria

Schulz, 1906

CLAVELLARIA Lamarck, 1801: misident.

#### Pseudoclavellaria amerinae

(Linnaeus, 1758)

Tenthredo amerinae Linnaeus, 1758
Tenthredo marginata (Linnaeus, 1767, *Tenthredo*)

##### Distribution

✝England

##### Notes

Extinct in Britain; reported as having been collected near Windsor (Leach 1817, Stephens 1835).

#### Trichiosoma

Leach, 1817

ASITRICHIOSOMA Malaise, 1937

##### Notes

Taxonomy follows Viitasaari (1985), Viitasaari (1989).

#### Trichiosoma laterale

Leach, 1817

Trichiosoma ?marginale Leach, 1817
Trichiosoma latreillei Leach, 1817

##### Distribution

England, Scotland, Ireland

#### Trichiosoma lucorum

(Linnaeus, 1758)

Tenthredo lucorum Linnaeus, 1758

##### Distribution

England, Scotland, Wales, Ireland

#### Trichiosoma pusillum

Stephens, 1835

Trichiosoma nigripes Gussakovskij, 1947

##### Distribution

England

#### Trichiosoma scalesii

Leach, 1817

Trichiosoma unidentatum Leach, 1817
Trichiosoma silvaticum Leach, 1817
Trichiosoma biverrucatum Stephens, 1835
Trichiosoma betuleti (Klug, 1834): Cameron, 1875 misident.

##### Distribution

England, Scotland

#### Trichiosoma sorbi

Hartig, 1840

Trichiosoma scalesii Leach, 1817: Cameron, 1885 misident.

##### Distribution

England, Scotland, Wales, Ireland

#### Trichiosoma tibiale

Stephens, 1835

Cimbex crataegi (Zaddach, 1863, *Cimbex*)

##### Distribution

England, Scotland, Wales, Ireland

#### Trichiosoma vitellina

(Linnaeus, 1760)

Tenthredo vitellina  Linnaeus, 1760
Trichiosoma boreale Gussakovskij, 1947
Trichiosoma vitellinae : misspelling

##### Distribution

England, Scotland, Ireland

#### Corynidinae

Benson, 1938

CORYNINAE preocc.

#### Corynis

Thunberg, 1789

AMASIS Leach, 1817

#### Corynis crassicornis

(Rossi, 1790)

Tenthredo crassicornis Rossi, 1790
Tenthredo laeta (Fabricius, 1798, *Tenthredo*)

##### Distribution

?England

##### Notes

Said to have been collected near Bristol (Leach 1817, Stephens 1835).

#### Corynis obscura

(Fabricius, 1775)

Tenthredo obscura  Fabricius, 1775

##### Distribution

?England

##### Notes

Said to have been collected in Lincolnshire (Leach 1817, Stephens 1835).

#### Diprionidae

Rohwer, 1910

#### Diprioninae

Rohwer, 1910

#### Diprion

Schrank, 1802

PTERONUS Jurine, 1801 suppressed
LOPHYRUS Latreille, 1802 suppressed

#### Diprion pini

(Linnaeus, 1758)

Diprion pini  *Tenthredo pini* Linnaeus, 1758

##### Distribution

England, Scotland, Wales, Ireland

#### Diprion similis

(Hartig, 1836)

Lophyrus similis  Hartig, 1836
Lophyrus eremita (Thomson, 1871, *Lophyrus*)

##### Distribution

England, Scotland, Wales

#### Gilpinia

Benson, 1939

#### Gilpinia frutetorum

(Fabricius, 1793)

Tenthredo frutetorum  Fabricius, 1793
Lophyrus thomsoni (Konow, 1884, *Lophyrus*)
Lophyrus variegatus Hartig, 1834: Cameron, 1890 misident.

##### Distribution

Scotland

#### Gilpinia hercyniae

(Hartig, 1837)

Lophyrus hercyniae  Hartig, 1837
Gilpinia polytoma (Hartig, 1834): misident.

##### Distribution

England, Wales

#### Gilpinia pallida

(Klug, 1812)

Lophyrus pallidus  Klug, 1812
Lophyrus virens (Klug, 1812): Cameron, 1885 misident.
Lophyrus dorsatus (Fabricius, 1781): Cameron, 1890 misident.

##### Distribution

England, Scotland

#### Gilpinia virens

(Klug, 1812)

Gilpinia virens  *Lophyrus virens* Klug, 1812

##### Distribution

England

#### Microdiprion

Enslin, 1917

#### Microdiprion pallipes

(Fallén, 1808)

Hylotoma pallipes  Fallén, 1808

##### Distribution

England, Scotland, Wales

#### Neodiprion

Rohwer, 1918

#### Neodiprion sertifer

(Geoffroy, 1785)

Tenthredo sertifera  Geoffroy, 1785
Neodiprion rufa (Retzius, 1783): Latreille, 1807 misident.

##### Distribution

England, Scotland, Wales, Ireland

#### Monocteninae

Benson, 1945

#### Monoctenus

Dahlbom, 1835

#### Monoctenus juniperi

(Linnaeus, 1758)

Tenthredo juniperi  Linnaeus, 1758

##### Distribution

Scotland, Ireland

#### Heptamelidae

Benson, 1938

##### Notes

Previously treated as a tribe of Selandriinae, raised to family rank by Malm and Nyman (2014) as the inclusion of *Heptamelus* and *Pseudoheptamelus* in the Tenthredinidae rendered the latter paraphyletic with respect to Cimbicidae and Diprionidae.

#### Heptamelus dahlbomi

(Thomson, 1870)

Caenoneura dahlbomi  Thomson, 1870
Heptamelus ochroleucus : misident.

##### Distribution

England, Scotland, Ireland

##### Notes

Added by Vikberg and Liston (2009).

#### Heptamelus ochroleucus

(Stephens, 1835)

Melicerta ochroleucus  Stephens, 1835

##### Distribution

England, Scotland, Wales, Ireland

#### Tenthredinidae

Latreille, 1802

##### Notes

Unplaced species of Tenthredinidae:

***Selandria ornata*** Newman, 1838

#### Allantinae

Rohwer, 1911

#### Allantini

Rohwer, 1911

#### Allantus

Panzer, 1801

EMPHYTUS Klug, 1815

##### Notes

According to Stephens (1835) (p.90), who gives a recognisable description of *Allantus didymus* (Klug, 1818), this species was “..rare: taken at Birch wood in June.” It is not included in the list of British species because no authenticated material has been located. A specimen without locality data in the Stephens Collection (NHM) is *Allantus melanarius* (det. G. Knight).

#### Allantus basalis

(Klug, 1818)

Tenthredo basalis Klug, 1818
Emphytus ?basalis ssp. caledonicus (Benson, 1945, *Emphytus*)

##### Distribution

England, Scotland

##### Notes

*Allantus basalis caledonicus* may represent a separate species (Blank and Taeger 1998) from Scottish specimens recorded as Allantus basalis ssp. basalis by Liston (1985) and similar specimens from northern England.

#### Allantus calceatus

(Klug, 1818)

Tenthredo calceata  Klug, 1818

##### Distribution

England, Scotland, Wales, Ireland

#### Allantus cinctus

(Linnaeus, 1758)

Tenthredo cincta  Linnaeus, 1758
Emphytus neglectus (Zaddach, 1859, *Emphytus*)
Emphytus cinctipes (Norton, 1867, *Emphytus*)

##### Distribution

England, Scotland, Wales, Ireland

#### Allantus cingulatus

(Scopoli, 1763)

Tenthredo cingulata  Scopoli, 1763

##### Distribution

England, Wales, Ireland

#### Allantus coryli

(Stritt, 1937)

Emphytus coryli  Stritt, 1937

##### Distribution

England

#### Allantus laticinctus

(Serville, 1823)

Dolerus laticinctus  Serville, 1823
Tenthredo balteatus (Klug, 1818, *Tenthredo*) preocc.

##### Distribution

Wales

##### Notes

Added by Knight (2006).

#### Allantus melanarius

(Klug, 1818)

Tenthredo melanaria  Klug, 1818

##### Distribution

England

#### Allantus rufocinctus

(Retzius, 1783)

Tenthredo rufocincta  Retzius, 1783

##### Distribution

England, Scotland, Wales, Ireland

#### Allantus togatus

(Panzer, 1801)

Allantus togatus  *Tenthredo togata* Panzer, 1801

##### Distribution

England, Scotland, Wales

#### Allantus truncatus

(Klug, 1818)

Tenthredo truncata  Klug, 1818
Allantus cingillum (Klug, 1818): Morice, 1909 misident.
Allantus melanarius (Klug, 1818): Morice, 1909 misident.

##### Distribution

England, Scotland, Wales

#### Apethymus

Benson, 1939

#### Apethymus filiformis

(Klug, 1818)

Tenthredo filiformis Klug, 1818
Tenthredo serotinus (Klug, 1818, *Tenthredo*) preocc.
Dolerus abdominalis (Serville, 1823, *Dolerus*)
Emphytus autumnalis (Forsius, 1933, *Emphytus*)

##### Distribution

England, Scotland, Wales, Ireland

#### Apethymus serotinus

(O. F. Müller, 1776)

Tenthredo serotina  Müller, 1776
Tenthredo braccatus (Gmelin, 1790, *Tenthredo*)
Tenthredo tibialis (Panzer, 1799, *Tenthredo*) preocc.
Emphytus panzeri (Kirby, 1882, *Emphytus*)

##### Distribution

England, Scotland, Wales, Ireland

#### Taxonus

Hartig, 1837

#### Taxonus agrorum

(Fallén, 1808)

Tenthredo agrorum  Fallén, 1808

##### Distribution

England, Scotland

#### Caliroini

Benson, 1938

#### Caliroa

Costa, 1859

ERIOCAMPOIDES Konow, 1890

#### Caliroa annulipes

(Klug, 1816)

Tenthredo annulipes  Klug, 1816
Selandria atra (Stephens, 1835, *Selandria*)

##### Distribution

England, Scotland, Wales, Ireland

#### Caliroa cerasi

(Linnaeus, 1758)

Tenthredo cerasi  Linnaeus, 1758
Tenthredo limacina (Retzius, 1783, *Tenthredo*)
Tenthredo adumbrata (Klug, 1816, *Tenthredo*)
Monostegia antipoda (W. F. Kirby, 1881, *Monostegia*)

##### Distribution

England, Scotland, Wales, Ireland

#### Caliroa cinxia

(Klug, 1816)

Tenthredo cinxia  Klug, 1816

##### Distribution

England

#### Caliroa tremulae

Chevin, 1974

Caliroa varipes (Klug, 1816): misident.

##### Distribution

England

##### Notes

Added by Liston (1993).

#### Caliroa varipes

(Klug, 1816)

Tenthredo varipes  Klug, 1816

##### Distribution

England, Wales, Ireland

#### Endelomyia

Ashmead, 1898

#### Endelomyia aethiops

(Gmelin, 1790)

Tenthredo aethiops  Gmelin, 1790
Selandria soror (Vollenhoven, 1869, *Selandria*)
Eriocampa testaceipes (Cameron, 1874, *Eriocampa*)
Eriocampa caninae (Cameron, 1878, *Eriocampa*)
Poecilosoma nigricolle (Cameron, 1882, *Poecilosoma*)

##### Distribution

England, Scotland, Wales, Ireland

#### Empriini

Rohwer, 1911

#### Ametastegia

Costa, 1882

PROTEMPHYTUS Rohwer, 1909
PROTOEMPHYTUS : misspelling

#### Ametastegia albipes

(Thomson, 1871)

Taxonus albipes  Thomson, 1871
Taxonus fletcheri (Cameron, 1878, *Taxonus*)
Emphytus nigricans (Klug, 1818): Stephens, 1835 misident.

##### Distribution

England, Wales, Ireland

#### Ametastegia carpini

(Hartig, 1837)

Emphytus carpini  Hartig, 1837
Taxonus glottianus (Cameron, 1874, *Taxonus*)

##### Distribution

England, Scotland, Wales, Ireland

##### Notes

*Taxonus glottianus* was justifiably synonymised with *Ametastegia tenera* by Konow (1899), because the original description strongly indicates the latter species, but after examination of the type Benson (1952) regarded *Taxonus glottianus* as a junior synonym of *Ametastegia carpini*. We follow Benson’s decision on the status of Cameron’s name.

#### Ametastegia equiseti

(Fallén, 1808)

Tenthredo equiseti  Fallén, 1808
Tenthredo coxalis (Hartig, 1837, *Tenthredo*)

##### Distribution

England, Scotland, Wales, Ireland

#### Ametastegia glabrata

(Fallén, 1808)

Tenthredo glabrata  Fallén, 1808

##### Distribution

England, Scotland, Wales, Ireland

#### Ametastegia pallipes

(Spinola, 1808)

Tenthredo pallipes  Spinola, 1808
Tenthredo grossulariae (Klug, 1818, *Tenthredo*)

##### Distribution

England, Scotland, Wales, Ireland

#### Ametastegia perla

(Klug, 1818)

Tenthredo perla  Klug, 1815

##### Distribution

England, Scotland, Ireland

#### Ametastegia tenera

(Fallén, 1808)

Tenthredo tenera  Fallén, 1808
Tenthredo patellata (Klug, 1818, *Tenthredo*)
Ametastegia tener : misspelling

##### Distribution

England, Scotland, Wales, Ireland

#### Empria

Lepeletier & Serville, 1828

POECILOSTOMA Dahlbom, 1835
POECILOSOMA Thomson, 1870 preocc.
PARATAXONUS MacGillivray, 1908
LEUCEMPRIA Enslin, 1913
TRIEMPRIA Enslin, 1914

##### Notes

Unplaced species of *Empria*:

[***Selandria signata*** (Newman, 1838, *Selandria*)]

#### Empria alector

Benson, 1938

##### Distribution

England, Scotland, Wales, Ireland

#### Empria alpina

Benson, 1938

Empria gussakovskii Dovnar-Zapolskij, 1929: Zhelochovtsev, 1988 misident.

##### Distribution

Scotland, Ireland

#### Empria basalis

Lindqvist, 1968

##### Distribution

England, Scotland

##### Notes

Added by Knight (2009).

#### Empria candidata

(Fallén, 1808)

Tenthredo candidata  Fallén, 1808
Tenthredo repanda (Klug, 1816, *Tenthredo*)

##### Distribution

England, Scotland, Wales

#### Empria excisa

(Thomson, 1871)

Poecilosoma excisa  Thomson, 1871

##### Distribution

England, Scotland, Wales, Ireland

#### Empria fletcheri

(Cameron, 1878)

Poecilosoma fletcheri  Cameron, 1878
Empria obtusa (Klug, 1817): Cameron, 1874 misident.

##### Distribution

Scotland

#### Empria immersa

(Klug, 1818)

Tenthredo immersa  Klug, 1818
Empria tirolensis Enslin, 1914

##### Distribution

England, Scotland, Wales, Ireland

#### Empria liturata

(Gmelin, 1790)

Tenthredo liturata  Gmelin, 1790

##### Distribution

England, Scotland, Wales, Ireland

#### Empria longicornis

(Thomson, 1871)

Poecilosoma longicornis  Thomson, 1871
Empria rubi Kontuniemi, 1951

##### Distribution

England, Scotland, Wales, Ireland

#### Empria minuta

Lindqvist, 1968

##### Distribution

Scotland

##### Notes

Added by Knight & Liston (in prep.).

#### Empria pallimacula

(Serville, 1823)

Dolerus pallimacula  Serville, 1823
Empria baltica Conde, 1937

##### Distribution

England, Scotland, Wales, Ireland

#### Empria parvula

(Konow, 1892)

Poecilosoma parvula  Konow, 1892

##### Distribution

England, Scotland

#### Empria pumila

(Konow, 1896)

Poecilosoma pumila  Konow, 1896

##### Distribution

England, Scotland, Wales, Ireland

##### Notes

*Empria pumiloides* Lindqvist, 1968 is distinguished from *Empria pumila* by Heidemaa and Viitasaari (1999). Although Scottish and Welsh specimens checked by Knight and Liston are all *Empria pumila*, the presence of *Empria pumiloides* in the British Isles cannot be ruled out.

#### Empria sexpunctata

(Serville, 1823)

Tenthredo sexpunctata  Serville, 1823
Selandria klugii (Stephens, 1835, *Selandria*)
Poecilosoma carbonaria (Konow, 1884, *Poecilosoma*)
Empria klugi : misspelling
Empria guttata (Fallén, 1808): Cameron, 1875 misident.

##### Distribution

England, Scotland, Wales, Ireland

#### Empria tridens

(Konow, 1896)

Poecilosoma tridens  Konow, 1896

##### Distribution

England, Scotland, Wales, Ireland

#### Harpiphorus

Hartig, 1837

ASTICTA Newman, 1838 preocc.

#### Harpiphorus lepidus

(Klug, 1818)

Tenthredo lepida  Klug, 1818
Fenusa ianthe (Newman, 1837, *Fenusa*)

##### Distribution

England, Wales, Ireland

#### Monostegia

Costa, 1859

NEMATOCEROS Konow, 1896

#### Monostegia abdominalis

(Fabricius, 1798)

Tenthredo abdominalis  Fabricius, 1798
Tenthredo luteola (Klug, 1816, *Tenthredo*)

##### Distribution

England, Wales, Ireland

#### Monsoma

MacGillivray, 1908

MONOSOMA Viereck, 1910

#### Monsoma pulveratum

(Retzius, 1783)

Tenthredo pulverata  Retzius, 1783
Monsoma pulverata : misspelling

##### Distribution

England, Scotland, Wales, Ireland

#### Eriocampini

Rohwer, 1911

#### Eriocampa

Hartig, 1837

BRACHYOCAMPA Zirngiebl, 1956

#### Eriocampa ovata

(Linnaeus, 1760)

Tenthredo ovata  Linnaeus, 1760

##### Distribution

England, Scotland, Wales, Ireland

#### Athaliinae

Rohwer, 1911

##### Notes

Raised to subfamily rank by Malm and Nyman (2014).

#### Athalia

Leach, 1817

DENTATHALIA Benson, 1931

#### Athalia ancilla

Serville, 1823

Athalia glabricollis Thomson, 1870

##### Distribution

England, Scotland, Wales

#### Athalia bicolor

Serville, 1823

Tenthredo annulata (Fabricius, 1787, *Tenthredo*) preocc.
Athalia richardi Serville, 1823

##### Distribution

England, Wales

#### Athalia circularis

(Klug, 1815)

Tenthredo circularis  Klug, 1815
Athalia lineolata Serville, 1823
Athalia ?cordatoides Kontuniemi, 1951

##### Distribution

England, Scotland, Wales, Ireland

##### Notes

There are grounds for suspecting that this consists of a group of species, with more narrowly defined host plant spectra than presently accepted.

#### Athalia cordata

Serville, 1823

##### Distribution

England, Scotland, Wales, Ireland

#### Athalia cornubiae

Benson, 1931

##### Distribution

England

#### Athalia liberta

(Klug, 1815)

Tenthredo liberta Klug, 1815

##### Distribution

England, Scotland, Wales, Ireland

#### Athalia lugens

(Klug, 1815)

Tenthredo lugens Klug, 1815

##### Distribution

England, Scotland, Wales, Ireland

#### Athalia rosae

(Linnaeus, 1758)

Tenthredo rosae  Linnaeus, 1758
Tenthredo colibri (Christ, 1791, *Tenthredo*)
Tenthredo spinarum (Fabricius, 1793, *Tenthredo*)
Tenthredo centifoliae (Panzer, 1797, *Tenthredo*)

##### Distribution

England, Scotland, Wales

#### Athalia scutellariae

Cameron, 1880

Dentathalia galericulatae (Kontuniemi, 1951, *Dentathalia*)

##### Distribution

England, Scotland, Wales, Ireland

#### Blennocampinae

Konow, 1890

#### Blennocampini

Konow, 1890

#### Ardis

Konow, 1886

#### Ardis pallipes

(Serville, 1823)

Dolerus pallipes  Serville, 1823
Tenthredo brunniventris (Hartig, 1837, *Tenthredo*)
Tenthredo bipunctata (Klug, 1817, *Tenthredo*) preocc.

##### Distribution

England, Scotland, Wales, Ireland

#### Ardis sulcata

(Cameron, 1882)

Blennocampa sulcata  Cameron, 1882

##### Distribution

England, Wales

#### Blennocampa

Hartig, 1837

#### Blennocampa phyllocolpa

Viitasaari & Vikberg, 1985

Tenthredo pusilla (Klug, 1816, *Tenthredo*) preocc.

##### Distribution

England, Scotland, Wales, Ireland

#### Cladardis

Benson, 1952

#### Cladardis elongatula

(Klug, 1817)

Tenthredo elongatula  Klug, 1817
Cladardis sericans (Hartig, 1837): Cameron, 1882 misident.

##### Distribution

?England

##### Notes

Presence in the British Isles uncertain: see Gibbs (2006).

#### Claremontia

Rohwer, 1909

PSEUDOBLENNOCAMPA Malaise, 1935

#### Claremontia alchemillae

(Cameron, 1877)

Claremontia alchemillae  *Blennocampa alchemillae* Cameron, 1877

##### Distribution

England, Scotland, Wales, Ireland

##### Notes

The taxonomy of this species, *Claremontia tenuicornis* (Klug, 1816) and *Claremontia uncta* (Klug, 1816) has not yet been fully resolved (Liston et al. 2006).

#### Claremontia alternipes

(Klug, 1816)

Tenthredo alternipes  Klug, 1816
Blennocampa intermedia (Kriechbaumer, 1884, *Blennocampa*)
Blennocampa tergestina (Kriechbaumer, 1888, *Blennocampa*)
Claremontia cinereipes (Klug, 1816): Thomson, 1870 misident.

#### Claremontia brevicornis

(Brischke, 1883)

Blennocampa brevicornis  Brischke, 1883
Blennocampa confusa (Konow, 1886, *Blennocampa*)
Claremontia geniculata (Hartig, 1837): Stephens, 1835 misident.
Claremontia alternipes (Klug, 1816): misident.

##### Distribution

England, Scotland, Wales, Ireland

#### Claremontia puncticeps

(Konow, 1886)

Claremontia puncticeps  *Blennocampa puncticeps* Konow, 1886

##### Distribution

England, Wales

#### Claremontia tenuicornis

(Klug, 1816)

Tenthredo tenuicornis  Klug, 1816
Selandria ?tibialis (Stephens, 1835, *Selandria*)
Blennocampa spiraeae (Brischke, 1883, *Blennocampa*)
Claremontia geniculata (Hartig, 1837): Enslin, 1918 misident.

##### Distribution

England, Scotland, Wales

##### Notes

Many previous records of this species in the British Isles seem to refer to *Claremontia alchemillae* and *Claremontia uncta*.

#### Claremontia uncta

(Klug, 1816)

Tenthredo uncta  Klug, 1816
Blennocampa humeralis (Vollenhoven, 1869, *Blennocampa*)

##### Distribution

England, Scotland, Ireland

##### Notes

Added by Koch (1988).

See above under *Claremontia alchemillae* / *Claremontia tenuicornis*.

#### Claremontia waldheimii

(Gimmerthal, 1847)

Tenthredo waldheimii  Gimmerthal, 1847
Selandria subcana (Zaddach, 1859, *Selandria*)
Blennocampa subserrata (Thomson, 1870, *Blennocampa*)

##### Distribution

England, Scotland, Wales

#### Monardis

Hartig, 1837

Tenthredo plana  Klug, 1817

#### Monardis plana

(Klug, 1817)

Tenthredo plana  Klug, 1817
Blennocampa rosarum (Brischke, 1883, *Blennocampa*)

##### Distribution

Wales

##### Notes

Added by Gibbs (2006).

#### Monophadnoides

Ashmead, 1898

PSEUDOMONOPHADNUS Malaise, 1935

#### Monophadnoides rubi

(T.W. Harris, 1845)

Selandria rubi  Harris, 1845
Tenthredo geniculata (Hartig, 1837, *Tenthredo*) preocc.

##### Distribution

England, Scotland, Wales, Ireland

#### Monophadnoides ruficruris

(Brullé, 1832)

Selandria ruficruris  Brullé, 1832

##### Distribution

England, Scotland, Wales

#### Pareophora

Konow, 1886

Tenthredo pruni  Linnaeus, 1758

#### Pareophora pruni

(Linnaeus, 1758)

Tenthredo pruni  Linnaeus, 1758
Tenthredo nigripes (Klug, 1816, *Tenthredo*) preocc.

##### Distribution

England, Ireland

#### Periclista

Konow, 1886

#### Periclista albida

(Klug, 1816)

Tenthredo albida  Klug, 1816
Tenthredo melanocephala (Fabricius, 1798, *Tenthredo*) preocc.
Selandria versicolor (Newman, 1837, *Selandria*)

##### Distribution

England, Scotland, Wales, Ireland

#### Periclista lineolata

(Klug, 1816)

Tenthredo lineolata  Klug, 1816

##### Distribution

England, Scotland

#### Periclista pubescens

(Zaddach, 1859)

Selandria pubescens  Zaddach, 1859

##### Distribution

England, Scotland, Wales

#### Fenusini

MacGillivray, 1906

#### Fenella

Westwood, 1840

PARAPHYLLOTOMA Forsius, 1930

#### Fenella monilicornis

(Dahlbom, 1835)

Phyllotoma monilicornis  Dahlbom, 1835
Fenella famosa Benson, 1950
Fenella minuta (Dahlbom, 1835): misident.

##### Distribution

Scotland

#### Fenella nigrita

Westwood, 1840

Phyllotoma tormentillae (Healy, 1868, *Phyllotoma*)
Fenella ?agrimoniae Brischke, 1888
Fenella pygmaea (Klug, 1816): Healy, 1869 misident.

##### Distribution

England, Scotland, Wales, Ireland

##### Notes

 Benson (1952) noted that biological differences possibly indicate the presence of two taxa under this name in the British Isles. This problem has still not been investigated.

#### Fenusa

Leach, 1817

KALIOSYSPHINGA Tischbein, 1846
APHADNURUS Costa, 1859
PHAENUSA Cameron, 1875
CALIOSYSPHINGA Konow, 1905

#### Fenusa dohrnii

(Tischbein, 1846)

Kaliosysphinga dohrnii  Tischbein, 1846
Phaenusa melanopoda (Cameron, 1876, *Phaenusa*)
Fenella westwoodi (Cameron, 1882, *Fenella*)

##### Distribution

England, Scotland, Wales, Ireland

#### Fenusa pumila

Leach, 1817

Tenthredo pumila (Klug, 1818, *Tenthredo*) preocc.
Fenusa pusilla (Lepeletier, 1823): misident.
Fenusa fuliginosa Healy, 1869
Fenusa minima Brischke, 1883
Fenusa pygmaea (Klug, 1816): Zetterstedt, 1838 misident.

##### Distribution

England, Scotland, Wales, Ireland

#### Fenusella

Enslin, 1914

MESSA Leach, 1817: misident.

#### Fenusella glaucopis

(Konow, 1907)

Fenusa glaucopis  Konow, 1907

##### Distribution

England, Scotland, Wales

#### Fenusella hortulana

(Klug, 1818)

Tenthredo hortulana  Klug, 1818
Fenusella soenderupi Hering, 1935

##### Distribution

England, Ireland

#### Fenusella nana

(Klug, 1816)

Tenthredo nana  Klug, 1816
Phyllotoma mellita (Newman, 1870, *Phyllotoma*)
Fenusa quercus (Cameron, 1885, *Fenusa*)
Scolioneura laeta (Enslin, 1918, *Scolioneura*)

##### Distribution

England, Scotland, Wales, Ireland

#### Heterarthrus

Stephens, 1835

PHYLLOTOMA Fallén, 1829 preocc.
DECATRIA Stephens, 1835
DRUIDA Newman, 1838
HETERARTHUS Cameron, 1882

##### Notes

Unplaced species of *Heterarthrus*:

[***Phyllotoma fumipennis*** (Cameron, 1888, *Phyllotoma*)]

#### Heterarthrus aceris

(Kaltenbach, 1856)

Phyllotoma aceris  Kaltenbach, 1856

##### Distribution

England, Scotland, Wales, Ireland

#### Heterarthrus cuneifrons

Altenhofer & Zombori, 1987

##### Distribution

England

##### Notes

Added by Liston and Blank (2006).

#### Heterarthrus microcephalus

(Klug, 1818)

Tenthredo microcephala  Klug, 1818
Heterarthrus melanopyga (Klug, 1818): Healy, 1868 misident.

##### Distribution

England, Scotland, Ireland

#### Heterarthrus nemoratus

(Fallén, 1808)

Hylotoma nemorata  Fallén, 1808
Fenusa parviceps (Newman, 1837, *Fenusa*)
Phyllotoma tenellus (Zaddach, 1859, *Phyllotoma*)
Phlebotrophia mathesoni (MacGillivray, 1909, *Phlebotrophia*)
Heterarthrus mathewsoni : misspelling

##### Distribution

England, Scotland, Wales, Ireland

#### Heterarthrus ochropoda

(Klug, 1818)

Tenthredo ochropoda  Klug, 1818
Decatria fuscipennis (Stephens, 1835, *Decatria*)
Phyllotoma pinguis (Vollenhoven, 1869, *Phyllotoma*)
Phyllotoma maxima (Strobl, 1896, *Phyllotoma*)
Heterarthrus ochropodus : misspelling

##### Distribution

England, Scotland, Wales

#### Heterarthrus vagans

(Fallén, 1808)

Hylotoma vagans  Fallén, 1808
Tenthredo melanopygus (Klug, 1818, *Tenthredo*)
Tenthredo amaurus (Klug, 1818, *Tenthredo*)
Phyllotoma kamtchaticus (Malaise, 1931, *Phyllotoma*)

##### Distribution

England, Scotland, Wales, Ireland

#### Heterarthrus wuestneii

(Konow, 1905)

Phyllotoma wuestneii  Konow, 1905
Phyllotoma aceris (McLachlan, 1867, *Phyllotoma*) preocc.
Heterarthrus healyi Altenhofer & Zombori, 1987

##### Distribution

England

##### Notes

Added by Altenhofer and Zombori (1987).

*Phyllotoma aceris* was described on the basis of specimens "bred by Mr Healy from larvae which make great blotches in the leaves of *Acer campestre*, and occasionally in *Acer pseudo-platanus*" McLachlan (1867). According to present knowledge on distribution and hostplants, the syntype series probably consisted of more than one species. The description is not sufficiently detailed to determine the identity of the specimens which McLachlan examined. No type material has been located, as already stated by Altenhofer and Zombori (1987). Nevertheless, Altenhofer and Zombori (1987) chose to regard *Phyllotoma aceris* McLachlan as being conspecific with a species which was reared from *Acer campestre* and proposed for it the new name *Heterarthrus healyi*. *Heterarthrus healyi* was subsequently found to be synonymous with *Heterarthrus wuestneii* (Konow, 1905) (Blank et al. 2001).

#### Kaliofenusa

MacGillivray, 1910

##### Notes

Unplaced species of *Kaliofenusa*:

[***Dolerus pusilla*** (Serville, 1823, *Dolerus*)]

#### Kaliofenusa altenhoferi

Liston, 1993

Kaliofenusa carpinifoliae Liston, 1993

##### Distribution

England, Scotland

##### Notes

Added by Liston (1994). Synonymy with *Kaliofenusa carpinifoliae* according to Liston (1996). *Dolerus pusillus* Serville cannot at present be placed as conspecific with *Kaliofenusa ulmi* or *Kaliofenusa altenhoferi*, and must be treated as an unplaced species (Blank et al. 2001).

#### Kaliofenusa ulmi

(Sundevall, 1847)

Fenusa ulmi  Sundevall, 1847
Fenusa intermedia (Thomson, 1871, *Fenusa*)
Messa alsia (MacGillivray, 1923, *Messa*)

##### Distribution

England, Scotland, Wales, Ireland

##### Notes

Recorded in Ireland by Knight (2004) as *Kaliofenusa pusilla*.

#### Metallus

Forbes, 1885

ENTODECTA Konow, 1886
POLYBATES MacGillivray, 1909

#### Metallus albipes

(Cameron, 1875)

Phaenusa albipes  Cameron, 1875
Entodecta tenuicornis (Hellén, 1935, *Entodecta*)

##### Distribution

England, Scotland

#### Metallus lanceolatus

(Thomson, 1870)

Blennocampa lanceolata  Thomson, 1870
Fenusa gei (Brischke, 1883, *Fenusa*)
Entodecta decolor (Konow, 1886, *Entodecta*)
Metallus bensoni Smith, 1971

##### Distribution

England, Scotland, Wales

#### Metallus pumilus

(Klug, 1816)

Tenthredo pumila  Klug, 1816
Emphytus pumilio (Hartig, 1837, *Emphytus*)
Fenusa rubi (Boie, 1848, *Fenusa*)

##### Distribution

England, Scotland, Ireland

#### Parna

Benson, 1936

#### Parna apicalis

(Brischke, 1888)

Blennocampa apicalis  Brischke, 1888
Parna reseri Liston, 1993

##### Distribution

England, Scotland, Wales

##### Notes

Added by Edmunds et al. (2007).

#### Parna tenella

(KIug, 1816)

Tenthredo tenella  Klug, 1816
Blennocampa tiliae (Kaltenbach, 1874, *Blennocampa*)

##### Distribution

England, Scotland, Wales

#### Profenusa

MacGillivray, 1914

#### Profenusa pygmaea

(Klug, 1816)

Tenthredo pygmaea  Klug, 1816

##### Distribution

England, Scotland, Wales, Ireland

#### Profenusa thomsoni

(Konow, 1886)

Fenusa thomsoni  Konow, 1886
Profenusa alumna (MacGillivray, 1923): misident.

##### Distribution

England

#### Scolioneura

Konow, 1890

#### Scolioneura betuleti

(Klug, 1816)

Tenthredo betuleti  Klug, 1816
Tenthredo nigricans (Klug, 1818, *Tenthredo*) preocc.
Fenusa betulae (Zaddach, 1859, *Fenusa*)
Scolioneura vicina Konow, 1894

##### Distribution

England, Scotland, Ireland

##### Notes

Synonymy of *Scolioneura vicina* with *Scolioneura betuleti* follows Leppänen et al. (2012).

#### Phymatocerini

Rohwer, 1911

TOMOSTETHINI Benson, 1938

#### Eutomostethus

Enslin, 1914

ATOMOSTETHUS Enslin, 1914

#### Eutomostethus ephippium

(Panzer, 1798)

Tenthredo ephippium  Panzer, 1798
Tenthredo dubius (Gmelin, 1790, *Tenthredo*) preocc.
Selandria inhabilis (Norton, 1861, *Selandria*)

##### Distribution

England, Scotland, Wales, Ireland

#### Eutomostethus gagathinus

(Klug, 1816)

Tenthredo gagathina  Klug, 1816

##### Distribution

England, Scotland, Wales

#### Eutomostethus luteiventris

(Klug, 1816)

Tenthredo luteiventris  Klug, 1816
Tenthredo fuscipennis (Serville, 1823, *Tenthredo*)

##### Distribution

England, Scotland, Wales, Ireland

#### Eutomostethus nigrans

(Konow, 1887)

Tomostethus nigrans  Konow, 1887
Eutomostethus nigrans Blank & Taeger, 1998 preocc.
Eutomostethus cinereipes (Klug, 1816): Cameron, 1882 misident.
Eutomostethus ephippium (Panzer, 1798): misident.

##### Distribution

England, Scotland, Wales, Ireland

##### Notes

Added by Liston and O'Connor (2005).

#### Eutomostethus punctatus

(Konow, 1887)

Tomostethus punctatus  Konow, 1887
Tomostethus brachycera (Cameron, 1893, *Tomostethus*)
Eutomostethus micans (Klug, 1816): Cameron, 1877 misident.

##### Distribution

England, Scotland, Wales

#### Monophadnus

Hartig, 1837

#### Monophadnus pallescens

(Gmelin, 1790)

Tenthredo pallescens  Gmelin, 1790
Tenthredo albipes (Gmelin, 1790, *Tenthredo*) preocc.
Monophadnus furvus Benson, 1930

##### Distribution

England, Scotland, Wales, Ireland

#### Paracharactus

MacGillivray, 1908

DICROSTEMA Benson, 1952

#### Paracharactus gracilicornis

(Zaddach, 1859)

Selandria gracilicornis  Zaddach, 1859

##### Distribution

England, Wales

#### Phymatocera

Dahlbom, 1835

PECTINIA Brullé, 1846
HYPARGYRICUS MacGillivray, 1908
PHYTOMATOCERA : misspelling

#### Phymatocera aterrima

(Klug, 1816)

Tenthredro aterrima  Klug, 1816
Selandria robinsoni (Curtis, 1850, *Selandria*)
Phymatocera fuliginosa (Schrank, 1781): misident.

##### Distribution

England, Scotland, Wales

#### Rhadinoceraea

Konow, 1886

#### Rhadinoceraea micans

(Schrank, 1781)

Tenthredo micans  Klug, 1816

##### Distribution

England, Wales

#### Stethomostus

Benson, 1939

#### Stethomostus fuliginosus

(Schrank, 1781)

Tenthredo fuliginosa  Schrank, 1781
Tenthredo fuscus (Serville, 1823, *Tenthredo*)

##### Distribution

England, Scotland, Wales

#### Stethomostus funereus

(Klug, 1816)

Tenthredo funerea  Klug, 1816

##### Distribution

England

#### Tomostethus

Konow, 1886

#### Tomostethus nigritus

(Fabricius, 1804)

Tenthredo nigrita  Fabricius, 1804

##### Distribution

England, Wales

#### Waldheimiini

Smith, 1969

#### Halidamia

Benson, 1939

#### Halidamia affinis

(Fallén, 1807)

Hylotoma affinis  Fallén, 1907
Tenthredo hyalina (Klug, 1816, *Tenthredo*)
Blennocampa assimilis (Thomson, 1870, *Blennocampa*)

##### Distribution

England, Scotland, Wales, Ireland

#### Nematinae

Thomson, 1871

##### Notes

Unplaced species of Nematinae:

***Nematus placidus*** Cameron, 1878

Placed by Konow (1905) as a synonym of *Pristiphora leucopodia* (Hartig, 1837), a species which has never been found in the British Isles. Konow probably did not examine the type of *Nematus placidus*, which according to Benson (1943b), is lost.

#### Cladiini

Ashmead, 1898

#### Cladius

Illiger, 1807

PRIOPHORUS Dahlbom, 1835
TRICHIOCAMPUS Hartig, 1837

#### Cladius brullei

(Dahlbom, 1835)

Priophorus brullei  Dahlbom, 1835
Cladius immunis Stephens, 1835
Nematus melanostigma (Stephens, 1835, *Nematus*)
Cladius tener Zaddach, 1859
Cladius tristis Zaddach, 1859
Cladius morio : misident.

##### Distribution

England, Scotland, Wales, Ireland

#### Cladius compressicornis

(Fabricius, 1804)

Tenthredo compressicornis  Fabricius, 1804
Tenthredo albipes (Fallén, 1808, *Tenthredo*) preocc.
Cladius pallipes Serville, 1823
Cladius padi : misident.

##### Distribution

England, Scotland, Wales, Ireland

##### Notes

The change in nomenclature is explained in Liston (2007).

#### Cladius grandis

(Serville, 1823)

Nematus grandis  Serville, 1823
Tenthredo viminalis (Fallén, 1808, *Tenthredo*) preocc.
Cladius luteicornis Stephens, 1835

##### Distribution

England, Scotland, Ireland

#### Cladius pectinicornis

(Geoffroy, 1785)

Tenthredo pectinicornis  Geoffroy, 1785
Tenthredo ?difformis (Panzer, 1799, *Tenthredo*)
Nematus crassicornis (Stephens, 1835, *Nematus*)

##### Distribution

England, Scotland, Wales, Ireland

#### Cladius pilicornis

Curtis, 1833

##### Distribution

England, Scotland, Wales, Ireland

#### Cladius rufipes

Serville, 1823

Cladius ulmi (Linnaeus, 1758): misident.

##### Distribution

England, Scotland, Ireland

#### Cladius ulmi

(Linnaeus, 1758)

Tenthredo ulmi  Linnaeus, 1759
Cladius eradiatus Hartig, 1837
Priophorus laevifrons (Benson, 1936, *Priophorus*)

##### Distribution

England, Scotland, Wales, Ireland

#### Dineurini

Ashmead, 1898

#### Anoplonyx

Marlatt, 1896

#### Anoplonyx destructor

Benson, 1952

Anoplonyx duplex (Lepeletier, 1823): misident.

##### Distribution

England, Scotland, Wales, Ireland

#### Dineura

Dahlbom, 1835

#### Dineura stilata

(Klug, 1816)

Tenthredo stilata  Klug, 1816
Nematus v-flavum (Cameron, 1882, *Nematus*)

##### Distribution

England, Scotland, Wales, Ireland

#### Dineura testaceipes

(Klug, 1816)

Tenthredo testaceipes  Klug, 1816

##### Distribution

England, Scotland, Wales, Ireland

#### Dineura virididorsata

(Retzius, 1783)

Tenthredo virididorsata  Retzius, 1783

##### Distribution

England, Scotland, Wales, Ireland

#### Hemichroa

Stephens, 1835

LEPTOCERA Hartig, 1837
LEPTOCERCUS Thomson, 1871

#### Hemichroa australis

(Serville, 1823)

Tenthredo australis  Serville, 1823
Tenthredo alni (Linnaeus, 1767, *Tenthredo*) preocc.
Tenthredo luctuosa (Hill, 1773, *Tenthredo*) nom. ob.

##### Distribution

England, Scotland, Wales, Ireland

#### Hemichroa crocea

(Geoffroy, 1785)

Tenthredo crocea  Geoffroy, 1785
Tenthredo rufa (Panzer, 1799, *Tenthredo*) preocc.
Hemichroa stigma Stephens, 1835

##### Distribution

England, Scotland, Wales, Ireland

#### Nematinus

Rohwer, 1911

##### Notes

Unplaced species of *Nematinus*:

[***Nematus antennatus*** (Cameron, 1877, *Nematus*)]

#### Nematinus acuminatus

(Thomson, 1871)

Nematus acuminatus  Thomson, 1871

##### Distribution

England, Scotland, Wales, Ireland

#### Nematinus caledonicus

(Cameron, 1882)

Nematus caledonicus  Cameron, 1882
Nematinus nigrosternatus Malaise, 1931

##### Distribution

England, Scotland, Ireland

#### Nematinus fuscipennis

(Serville, 1823)

Nematus fuscipennis  Serville, 1823
Nematinus abdominalis (Fabricius, 1798): misident.

##### Distribution

England, Scotland, Wales, Ireland

#### Nematinus luteus

(Panzer, 1803)

Nematus luteus  Panzer, 1803
Nematinus willigkiae R. Stein, 1926
Nematinus willigkiae ssp. pilosus Benson, 1958

##### Distribution

England, Scotland, Wales, Ireland

#### Nematinus steini

Blank, 1998

Tenthredo alneti (Bechstein & Scharfenberg, 1805, *Tenthredo*) preocc.
Nematinus bilineatus (Klug, 1819): Cameron, 1877, misident.
Nematinus ruficapillus (Gmelin, 1790): Kirby, 1882, misident.
Nematinus luteus (Panzer, 1803): misident.

##### Distribution

England, Scotland, Wales, Ireland

#### Platycampus

Schiødte, 1839

LEPTOPUS Hartig, 1837 preocc.
CAMPONISCUS Newman, 1869

#### Platycampus luridiventris

(Fallén, 1808)

Tenthredo luridiventris  Fallén, 1808
Nematus niger (Stephens, 1835, *Nematus*)
Nematus alnivorus (Hartig, 1840, *Nematus*)
Camponiscus healaei (Newman, 1869, *Camponiscus*)

##### Distribution

England, Scotland, Wales, Ireland

#### Hoplocampini

Konow, 1890

#### Hoplocampa

Hartig, 1837

#### Hoplocampa alpina

(Zetterstedt, 1838)

Tenthredo alpina  Zetterstedt, 1838
Selandria pallida (Newman, 1837, *Selandria*) preocc.

##### Distribution

England, Scotland, Ireland

#### Hoplocampa ariae

Benson, 1933

##### Distribution

England, Ireland

#### Hoplocampa brevis

(Klug, 1816

Tenthredo brevis  Klug, 1816

##### Distribution

England

#### Hoplocampa chrysorrhoea

(Klug, 1816)

Tenthredo chrysorrhoea  Klug, 1816

##### Distribution

England, Scotland, Wales, Ireland

#### Hoplocampa crataegi

(Klug, 1816)

Tenthredo crataegi  Klug, 1816
Hoplocampa plagiata (Klug, 1816): Cameron, 1885 misident.

##### Distribution

England, Scotland, Wales, Ireland

#### Hoplocampa flava

(Linnaeus, 1761)

Tenthredo flava  Linnaeus, 1760
Hoplocampa ferruginea (Fabricius, 1804): misident.
Hoplocampa minuta (Christ, 1791): misident.

##### Distribution

England, Scotland, Wales, Ireland

#### Hoplocampa fulvicornis

(Panzer, 1801)

Tenthredo fulvicornis  Panzer, 1801
Tenthredo rutilicornis (Klug, 1816, *Tenthredo*)

##### Distribution

England, Scotland, Wales, Ireland

#### Hoplocampa pectoralis

Thomson, 1871

Hoplocampa gallicola Cameron, 1877

##### Distribution

England, Scotland, Wales, Ireland

#### Hoplocampa testudinea

(Klug, 1816)

Tenthredo testudinea  Klug, 1816

##### Distribution

England, Scotland, Wales

#### Mesoneurini

Zombori, 1982

#### Mesoneura

Hartig, 1837

#### Mesoneura opaca

(Fabricius, 1775)

Tenthredo opaca  Fabricius, 1775
Tenthredo verna (Klug, 1816, *Tenthredo*)
Selandria biloba (Stephens, 1835, *Selandria*)
Dineura selandriiformis (Cameron, 1875, *Dineura*)

##### Distribution

England, Scotland, Wales, Ireland

#### Nematini

Thomson, 1871

#### Amauronematus

Konow, 1890

PONTOPRISTIA Malaise, 1921
BRACHYCOLUMA Strand, 1929
BRACHYCOLUS Konow, 1895 preocc.
DECANEMATUS Malaise, 1931

#### Amauronematus abnormis

(Holmgren, 1883)

Nematus abnormis  Holmgren, 1883
Amauronematus tolli Konow, 1907
Amauronematus aulatus MacGillivray, 1919

##### Distribution

Scotland

#### Amauronematus amentorum

(Förster, 1854)

Nematus amentorum  Förster, 1854
Nematus suavis (Ruthe, 1859, *Nematus*)
Pontopristia kamtchaticus (Malaise, 1931, *Pontopristia*)

##### Distribution

England, Scotland

#### Amauronematus amplus

Konow, 1895

##### Distribution

England, Scotland, Ireland

#### Amauronematus fasciatus

Konow, 1897

Amauronematus perkinsi Benson, 1933
Amauronematus variabilis Malaise, 1931: misident.

##### Distribution

England, Scotland, Wales, Ireland

#### Amauronematus godmani

Benson, 1955

##### Distribution

Scotland

##### Notes

Specimens identified by Benson as this species in NMS, from Switzerland (type locality) and Scotland, do not seem to be conspecific. They both belong to the *Amauronematus variator* (Ruthe) species group. The identity of Scottish specimens requires further study.

#### Amauronematus hedstroemi

Malaise, 1931

Amauronematus rex Benson, 1948
Amauronematus tillbergi Malaise, 1920: misident.

##### Distribution

England, Scotland, Wales, Ireland

##### Notes

See Lindqvist (1972) on taxonomy of this species and *Amauronematus tillbergi* Malaise, 1920.

#### Amauronematus histrio

(Serville, 1823)

Nematus histrio  Serville, 1823
Nematus rufescens (Hartig 1837, *Nematus*)
Nematus glenelgensis (Cameron, 1882, *Nematus*)

##### Distribution

England, Scotland, Ireland

##### Notes

British and Irish specimens at present identified as this species or as *Amauronematus stenogaster* may include other taxa not yet recorded in the British Isles. See revision of North European taxa by Schmidt (1997).

#### Amauronematus humeralis

(Serville, 1823)

Nematus humeralis  Serville, 1823
Amauronematus terminalis Malaise, 1931

##### Distribution

England, Scotland, Wales

#### Amauronematus krausi

Taeger & Blank, 1998

Amauronematus puniceus (Christ, 1791): misident.

##### Distribution

England

#### Amauronematus lateralis

Konow, 1896

Amauronematus trautmanni Enslin, 1919
Amauronematus cameroni Perkins, 1929
Amauronematus piliserra Lindqvist, 1943
Amauronematus imperfectus (Zaddach, 1876): Cameron, 1885 misident.

##### Distribution

England, Scotland, Wales, Ireland

#### Amauronematus leucolenus

(Brischke, 1883)

Nematus leucolenus Brischke, 1883
Amauronematus saarineni (Lindqvist, 1935): Benson, 1948 misident.
Amauronematus leucolaenus : Benson, 1958 misspelling

##### Distribution

England, Ireland

#### Amauronematus longiserra

(Thomson, 1863)

Nematus longiserra  Thomson, 1863

##### Distribution

England, Scotland, Wales

#### Amauronematus mcluckieae

Benson, 1935

Amauronematus pustulatus Lindqvist, 1962
Amauronematus arcticola Enslin, 1915: misident.

##### Distribution

Scotland

##### Notes

See Liston et al. (2012) on the spelling of the species name.

#### Amauronematus miltonotus

(Zaddach, 1883)

Nematus miltonotus  Zaddach, 1883

##### Distribution

England, Wales

#### Amauronematus mimus

Schmidt, 1997

##### Distribution

Scotland

##### Notes

Added by Liston et al. (2012).

#### Amauronematus mundus

Konow, 1895

##### Distribution

England, Scotland, Wales, Ireland

#### Amauronematus sagmarius

Konow, 1895

##### Distribution

England, Scotland, Ireland

#### Amauronematus semilacteus

(Zaddach, 1883)

Nematus semilacteus  Zaddach, 1883

##### Distribution

Scotland

#### Amauronematus stenogaster

(Förster, 1854)

Nematus stenogaster  Förster, 1854
Amauronematus festivus Saarinen, 1950
Amauronematus fallax (Serville, 1823): misident.

##### Distribution

England, Scotland, Wales, Ireland

#### Amauronematus toeniatus

(Serville, 1823)

Nematus toeniatus  Serville, 1823
Nematus taeniatus (Lepeletier, 1823, *Nematus*) preocc.
Amauronematus aemulus Konow, 1895
Amauronematus zetterstedti Malaise, 1920
Amauronematus alpicola Konow, 1895: misident.

##### Distribution

England, Scotland

#### Amauronematus tristis

Lindqvist, 1959

Amauronematus sempersolis Kiaer, 1898: misident.

##### Notes

Added by Knight & Liston (in prep.).

#### Amauronematus tunicatus

(Zaddach, 1883)

Nematus tunicatus  Zaddach, 1883

##### Distribution

England, Ireland

#### Amauronematus viduatus

(Zetterstedt, 1838)

Tenthredo viduata  Zetterstedt, 1838
Nematus notatus (Förster, 1854, *Nematus*)
Amauronematus longiserra (Thomson, 1863): Cameron, 1876 misident.

##### Distribution

England, Scotland, Wales, Ireland

#### Amauronematus vittatus

(Serville, 1823)

Nematus vittatus  Serville, 1823
Amauronematus crispus Benson, 1948

##### Distribution

England, Scotland, Wales, Ireland

#### Craesus

Leach, 1817

CROESUS Curtis, 1824: misspelling

#### Craesus alniastri

(Scharfenberg, 1805)

Tenthredo alniastri  Scharfenberg, 1805
Nematus varus (Villaret, 1832, *Nematus*)

##### Distribution

England, Scotland, Wales, Ireland

#### Craesus brischkei

(Zaddach, 1876)

Nematus brischkei  Zaddach, 1876

##### Distribution

England

#### Craesus latipes

(Villaret, 1832)

Nematus latipes  Villaret, 1832

##### Distribution

England, Scotland, Wales, Ireland

#### Craesus septentrionalis

(Linnaeus, 1758)

Tenthredo septentrionalis  Linnaeus, 1758
Craesus stephensii Newman, 1837

##### Distribution

England, Scotland, Wales, Ireland

#### Euura

Newman, 1837

CRYPTOCAMPUS Hartig, 1837
GEMMURA Smith, 1968

#### Euura amerinae

(Linnaeus, 1758)

Cynips amerinae  Linnaeus, 1758
Tenthredo salicispentandrae (Retzius, 1783, *Tenthredo*)
Nematus medullarius (Hartig, 1837, *Nematus*)
Tenthredo saliceti (Fallén, 1808, *Tenthredo*)
Euura mucronata (Hartig, 1837): Vollenhoven, 1871 misident.

##### Distribution

England, Scotland, Ireland

#### Euura atra

(Jurine, 1807)

Pteronus ater  Jurine, 1807

##### Distribution

England, Scotland, Wales, Ireland

##### Notes

Regarded as a group of sibling (biological) species by Kopelke (1996), Kopelke (2000). These are very difficult, perhaps impossible, to distinguish using adult morphological characters. According to records of galls on various identified *Salix* species, at least the following three species belonging to the group occur in the British Isles: *Euura auritae*, *Euura purpureae* and *Euura weiffenbachii*.

#### Euura auritae

Kopelke, 2000

##### Distribution

Scotland

#### Euura mucronata

(Hartig, 1837)

Nematus mucronatus  Hartig, 1837
Euura gallae Newman, 1837
Cryptocampus ?nigritarsis (Cameron, 1885, *Cryptocampus*)
Euura saliceti (Fallén, 1808): misident.

##### Distribution

England, Scotland, Wales, Ireland

##### Notes

According to Kopelke (2001), several biological species are included under this name. A number of these seem to occur in the British Isles, but an alternative view on some of the taxa proposed by Kopelke has been offered by Nyman (2002).

#### Euura purpureae

Kopelke, 1996

##### Distribution

Ireland

##### Notes

The only known record in the British Isles is based on the specimens mentioned by O’Connor et al. (1997) under the name *Euura atra*, as having been reared from *Salix purpurea*.

#### Euura testaceipes

(Brischke, 1883)

Cryptocampus testaceipes  Brischke, 1883
Euura cynips Newman, 1837 nom. ob.

##### Distribution

England, Scotland

#### Euura venusta

(Brischke, 1883)

Cryptocampus venustus  Brischke, 1883

##### Distribution

England, Scotland, Wales

#### Euura weiffenbachii

Ermolenko, 1988

##### Distribution

Scotland, Ireland

##### Notes

Added by Liston and O'Connor (2005).

#### Nematus

Panzer, 1801

#### Kontuniemiana

Lacourt, 1998

#### Nematus (Kontuniemiana) leucotrochus

Hartig, 1837

Nematus maculiventris Hartig, 1840
Nematus approximatus Förster, 1854
Nematus consobrinus Vollenhoven, 1871
Pteronidea cognatus (Lindqvist, 1957, *Pteronidea*)

##### Distribution

England, Scotland, Wales, Ireland

#### Nematus (Kontuniemiana) olfaciens

Benson, 1953

##### Distribution

England, Scotland

#### Nematus (Kontuniemiana) ribesii

(Scopoli, 1763)

Tenthredo ribesii  Scopoli, 1763
Nematus dimidiatus Serville, 1823
Nematus trimaculatus Serville, 1823
Nematus grossulariae Moore, 1831
Tenthredo ventricosus (Bouché, 1834, *Tenthredo*)
Nematus macrocerus Hartig, 1840
Nematus ribis Dufour, 1847

##### Distribution

England, Scotland, Wales, Ireland

#### Nematus

Panzer, 1801

HYPOLAEPUS W. F. Kirby, 1882
HOLCOCNEME Konow, 1890

#### Nematus (Nematus) caeruleocarpus

Hartig, 1837

Nematus brevispinis Förster, 1854
Nematus brachyacanthus Thomson, 1863
Nematus sulcipes Hartig, 1837
Nematus coeruleocarpus : misspellling

##### Distribution

England, Scotland, Ireland

#### Nematus (Nematus) lucidus

(Panzer, 1801)

Tenthredo lucida  Panzer, 1801

##### Distribution

England, Scotland, Wales, Ireland

#### Nematus (Nematus) vicinus

Serville, 1823

Nematus longispinus Kriechbaumer, 1885
Tenthredo crassus (Fallén, 1808, *Tenthredo*) preocc.
Nematus sulcipes (Hartig, 1837): misident.

##### Distribution

England, Scotland, Wales, Ireland

#### Pteronidea

Rohwer, 1911

LINDQVISTIA Lacourt, 1998

#### Nematus (Pteronidea) bergmanni

Dahlbom, 1835

Nematus pallicarpus Hartig, 1837
Nematus curtispina Thomson, 1871
Lygaeonematus pallens (Enslin, 1916, *Lygaeonematus*)
Pteronidea vernalis (Lindqvist, 1937, *Pteronidea*)

##### Distribution

England, Scotland, Wales, Ireland

#### Nematus (Pteronidea) bipartitus

Serville, 1823

##### Distribution

England, Scotland, Wales

#### Nematus (Pteronidea) brevivalvis

Thomson, 1871

Pteronus kriegeri (Konow, 1903, *Pteronus*)
Amauronematus spurcus (Konow, 1904, *Amauronematus*)
Pteronidea absimilis (Lindqvist, 1939, *Pteronidea*)

##### Distribution

England, Scotland, Wales, Ireland

#### Nematus (Pteronidea) cadderensis

Cameron, 1875

Pteronidea macroserratus (Lindqvist, 1943, *Pteronidea*)

##### Distribution

England, Scotland, Ireland

#### Nematus (Pteronidea) dorsatus

Cameron, 1875

##### Distribution

Scotland, Wales

#### Nematus (Pteronidea) fagi

Zaddach, 1876

##### Distribution

England, Scotland, Wales

#### Nematus (Pteronidea) fahraei

Thomson, 1862

##### Distribution

England, Scotland

#### Nematus (Pteronidea) ferrugineus

Förster, 1854

Nematus glottianus Cameron, 1882

##### Distribution

England, Scotland, Ireland

#### Nematus (Pteronidea) flavescens

Stephens, 1835

##### Distribution

England, Scotland, Wales, Ireland

#### Nematus (Pteronidea) frenalis

Thomson, 1888

Pteronus fastosus (Konow, 1904, *Pteronus*)
Nematus fastuosus : misspelling

##### Distribution

Scotland, Ireland

#### Nematus (Pteronidea) fuscomaculatus

Förster, 1854

Nematus strongylogaster Cameron, 1878
Pteronus dossuarius (Konow, 1904, *Pteronus*)

##### Distribution

England, Scotland

#### Nematus (Pteronidea) hypoxanthus

Förster, 1854

Nematus orbitalis Cameron, 1884
Pteronidea nigronotus (Lindqvist, 1957, *Pteronidea*)

##### Distribution

England, Scotland, Wales, Ireland

#### Nematus (Pteronidea) incompletus

Förster, 1854

Nematus pulchellus Cameron, 1882
Nematus segmentarius Förster, 1854: misident.

##### Distribution

England, Scotland, Ireland

#### Nematus (Pteronidea) jugicola

Thomson, 1871

Pteronidea karvoneni (Lindqvist, 1969, *Pteronidea*)

##### Distribution

England, Scotland

#### Nematus (Pteronidea) leionotus

(Benson, 1933)

Pteronidea leionota  Benson, 1933

##### Distribution

England, Scotland, Ireland

#### Nematus (Pteronidea) melanocephalus

Hartig, 1837

##### Distribution

England, Scotland, Wales, Ireland

#### Nematus (Pteronidea) miliaris

(Panzer, 1797)

Tenthredo miliaris  Panzer, 1797
Nematus capreae (Linnaeus, 1758): misident.
Nematus croceus (Fallén, 1808): misident.
Nematus testaceus Stephens, 1835

##### Distribution

England, Scotland, Wales, Ireland

#### Nematus (Pteronidea) monticola

Thomson, 1871

Pachynematus perkioemaekii (Lindqvist, 1960, *Pachynematus*)
Nematus ?pschornwalcheri Muche, 1972
Nematus similator Förster, 1854: misident.

##### Distribution

England, Scotland

#### Nematus (Pteronidea) myosotidis

(Fabricius, 1804)

Tenthredo myosotidis  Fabricius, 1804
Nematus ambiguus Förster, 1854
Nematus papillosus (Retzius, 1783): misident.

##### Distribution

England, Scotland, Wales, Ireland

#### Nematus (Pteronidea) nigricornis

Serville, 1823

##### Distribution

England, Scotland, Wales, Ireland

#### Nematus (Pteronidea) nubium

(Benson, 1935)

Pteronidea nubium  Benson, 1935
Pteronidea roberti (Lindqvist, 1958, *Pteronidea*)

##### Distribution

Scotland

#### Nematus (Pteronidea) oligospilus

Förster, 1854

Nematus salicivorus Cameron, 1882
Nematus miliaris (Panzer, 1797): Cameron, 1885 misident.
Nematus capreae (Linnaeus, 1758): misident.

##### Distribution

England, Scotland, Wales, Ireland

#### Nematus (Pteronidea) papillosus

(Retzius, 1783)

Tenthredo papillosa  Retzius, 1783
Nematus melanaspis Hartig, 1840
Nematus ?maculiger Cameron, 1882
Pteronidea sveae (Lindqvist, 1958, *Pteronidea*)

##### Distribution

England, Scotland, Wales, Ireland

#### Nematus (Pteronidea) pavidus

Serville, 1823

Nematus wttewaalli Vollenhoven, 1862
Nematus cameronii Dalla Torre, 1894
Nematus aurantiacus Hartig, 1837: misident.
Nematus similator Förster, 1854: misident.

##### Distribution

England, Scotland, Wales, Ireland

#### Nematus (Pteronidea) poecilonotus

Zaddach, 1876

Nematus viridescens Cameron, 1885
Pteronidea subnitens (Lindqvist, 1957, *Pteronidea*)
Nematus palliatus Thomson, 1863: misident.

##### Distribution

England, Scotland, Wales, Ireland

#### Nematus (Pteronidea) pravus

(Konow, 1895)

Amauronematus pravus  Konow, 1895

##### Distribution

Scotland

##### Notes

Added by Knight and Liston (in prep.). Some differences between Scottish individuals and the Estonian lectotype require further investigation.

#### Nematus (Pteronidea) pseudodispar

(Lindqvist, 1969)

Pteronidea pseudodispar  Lindqvist, 1969

##### Distribution

England, Scotland

##### Notes

Added by Knight and Liston (in prep.).

#### Nematus (Pteronidea) reticulatus

Holmgren, 1883

Nematus arcticus Thomson, 1871 preocc.
Nematus arcticola Dalla Torre, 1894
Pontania forsiusi (Enslin, 1915, *Pontania*)
Amauronematus alsius (Benson, 1935, *Amauronematus*)

##### Distribution

Scotland

#### Nematus (Pteronidea) salicis

(Linnaeus, 1758)

Tenthredo salicis  Linnaeus, 1758

##### Distribution

England, Scotland, Wales

#### Nematus (Pteronidea) spiraeae

Zaddach, 1883

##### Distribution

England, Scotland, Wales, Ireland

#### Nematus (Pteronidea) stichi

(Enslin, 1913)

Pteronidea stichi  Enslin, 1913
Nematus testaceus Thomson, 1871 preocc.
Pteronidea fuscarima (Benson, 1933, *Pteronidea*)
Nematus pallescens Hartig, 1837: Cameron, 1877 misident.

##### Distribution

England, Scotland, Ireland

#### Nematus (Pteronidea) sylvestris

Cameron, 1884

Nematus punctiscuta Hellén, 1948
Nematus ponojensis Hellén, 1948
Nematus silvestris : misspelling

##### Distribution

England, Scotland

#### Nematus (Pteronidea) tibialis

Newman, 1837

Nematus hortensis Hartig, 1837
Nematus trilineatus Norton, 1867
Nematus similaris Norton, 1880
Nematus robiniae Forbes, 1885

##### Distribution

England

#### Nematus (Pteronidea) umbratus

Thomson, 1871

Nematus collinus Cameron, 1882
Pteronidea similis (Forsius, 1911, *Pteronidea*)
Pteronidea verrucosae (Kontuniemi, 1966, *Pteronidea*)

##### Distribution

England, Scotland, Ireland

#### Nematus (Pteronidea) viridis

Stephens, 1835

Nematus prasinus Hartig, 1837
Nematus brevivalvis Thomson, 1871: Konow, 1890 misident.
Nematus dispar Zaddach, 1876: misident.
Nematus bergmanni Dahlbom, 1835: misident.

##### Distribution

England, Scotland, Wales, Ireland

#### Nematus (Pteronidea) viridissimus

Möller, 1882

Nematus ?glutinosae Cameron, 1882
Nematus polyspilus Förster, 1854: misident.

##### Distribution

England, Scotland, Wales, Ireland

##### Notes

According to Lindqvist (1962), *Nematus glutinosae* (description probably predates that of *Nematus viridissimus* by a few months) is not conspecific with *Nematus viridissimus*.

#### Pachynematus

Konow, 1890

PIKONEMA Ross, 1937
LARINEMATUS Zhelochovtsev, 1988
POLYNEMATUS Zhelochovtsev, 1988
EPICENEMATUS Lacourt, 1998

#### Pachynematus albipennis

(Hartig, 1837)

Nematus albipennis  Hartig, 1837
Pachynematus sannio (Konow, 1903, *Pachynematus*)

##### Distribution

England, Scotland, Wales, Ireland

#### Pachynematus annulatus

(Gimmerthal, 1834)

Nematus annulatus  Gimmerthal, 1834
Nematus flavipennis (Cameron, 1876, *Nematus*)
Pachynematus arcticus (Thomson, 1871): Cameron, 1878 misident.
Pachynematus rumicis (Linnaeus, 1758): Fallén, 1808 misident.
Pachynematus rumicis : Benson, 1958

##### Distribution

England, Scotland, Ireland

#### Pachynematus calcicola

Benson, 1948

Pachynematus chambersi Benson, 1948
Pachynematus laevigatus Zaddach, 1883: Benson, 1967 misident.

##### Distribution

England, Ireland

#### Pachynematus clibrichellus

(Cameron, 1882)

Nematus clibrichellus  Cameron, 1878
Nematus thomsoni (Cameron, 1882, *Nematus*)
Nematus clibrichensis (Cameron, 1885, *Nematus*): unjustified emendation
Pachynematus hyperboreus (Thomson, 1871): Cameron, 1878 misident.

##### Distribution

England, Scotland, Wales

#### Pachynematus clitellatus

(Serville, 1823)

Nematus clitellatus  Serville, 1823
Nematus trisignatus (Förster, 1854, *Nematus*)
Pachynematus foveolatus Konow, 1903
Pachynematus truncatus Benson, 1948
Pachynematus extensicornis (Norton, 1861): misident.

##### Distribution

England, Scotland, Wales, Ireland

##### Notes

Taeger and Blank (1998) regard *Pachynematus clitellatus* and *Pachynematus fallax* as morphologically highly variable species, which together include most of the previously described W. Palaearctic taxa. Their arguments for this are well reasoned, but at present it seems prudent to continue to regard several of the more distinctive morphological segregates, treated as species by Benson (1958), as separate taxa, because morphologically intermediate specimens are rare (at least in the male sex). The taxonomy of the whole group requires further intensive work, including genetic analysis. According to Taeger and Blank (1998) *Pachynematus kirbyi*, treated here as valid, is a synonym of *Pachynematus clitellatus*.

#### Pachynematus fallax

(Serville, 1823)

Nematus fallax  Serville, 1823
Nematus xanthocarpus (Hartig, 1840, *Nematus*)
Pachynematus virginalis Liston, 1982

##### Distribution

England, Scotland, Ireland

##### Notes

See above, under *Pachynematus clitellatus*. According Taeger and Blank (1998), *Pachynematus sulcatus* and *Pachynematus calcicola* are synonyms of *Pachynematus xanthocarpus* (= *Pachynematus fallax*).

#### Pachynematus glabriceps

Lindqvist, 1949

Pachynematus parvilabris (Thomson, 1863): misident.

##### Distribution

England

#### Pachynematus imperfectus

(Zaddach, 1876)

Nematus imperfectus  Zaddach, 1876

##### Distribution

England, Scotland, Wales

#### Pachynematus kirbyi

(Dahlbom, 1835)

Nematus kirbyi  Dahlbom, 1835
Nematus flaviventris (Hartig, 1840, *Nematus*)
Nematus melanocerus (Hartig, 1840, *Nematus*)
Nematus diaphanus (Eversmann, 1847, *Nematus*)
Nematus umbripennis (Eversmann, 1847, *Nematus*)
Nematus turgidus (Zaddach, 1876, *Nematus*)

##### Distribution

England, Scotland, Wales, Ireland

#### Pachynematus lichtwardti

Konow, 1903

Nematus apicalis (Hartig, 1837, *Nematus*) preocc.

##### Distribution

England, Scotland, Wales, Ireland

#### Pachynematus moerens

(Förster 1854)

Nematus moerens  Förster, 1854
Nematus pygostolus (Förster, 1854, *Nematus*)
Nematus pleuralis (Thomson, 1863, *Nematus*)
Pachynematus pullus Konow, 1903
Pachynematus torridonensis Liston, 1980

##### Distribution

England, Scotland, Wales, Ireland

#### Pachynematus montanus

(Zaddach, 1883)

Nematus montanus  Zaddach, 1883

##### Distribution

England, Scotland, Wales, Ireland

#### Pachynematus obductus

(Hartig, 1837)

Nematus obductus  Hartig, 1837
Nematus conductus (Ruthe, 1859, *Nematus*)
Nematus graminis (Cameron, 1874, *Nematus*)

##### Distribution

England, Scotland, Wales, Ireland

#### Pachynematus scutellatus

(Hartig, 1837)

Nematus scutellatus  Hartig, 1837
Pikonema scutellatum : Lacourt, 1999

##### Distribution

England, Scotland, Wales, Ireland

#### Pachynematus smithae

Ross, 1945

Pachynematus angustatus Lindqvist, 1949
Pachynematus smithiae : misspelling

##### Distribution

England, Scotland

#### Pachynematus sulcatus

Benson, 1948

##### Distribution

England, Scotland

#### Pachynematus vagus

(Fabricius, 1781)

Tenthredo vaga  Fabricius, 1781
Nematus leucogaster (Hartig, 1840, *Nematus*)
Nematus inconspicuus (W. F. Kirby, 1882, *Nematus*)

##### Distribution

England, Scotland, Wales, Ireland

#### Phyllocolpa

Benson, 1960

#### Phyllocolpa acutiserra

(Lindqvist, 1949)

Pontania acutiserra  Lindqvist, 1949

##### Distribution

Scotland

#### Phyllocolpa alienata

(Förster, 1854)

Nematus alienatus  Förster, 1854
Phyllocolpa coriacea (Benson, 1953): misident.

##### Distribution

Scotland

#### Phyllocolpa anglica

(Cameron, 1877

Nematus anglicus  Cameron, 1877
Nematus nigrolineata (Cameron, 1879, *Nematus*)

##### Distribution

England, Ireland

##### Notes

Three morphologically well distinguished *Phyllocolpa* species feed on *Salix viminalis* in Europe (Vikberg 2010a). Benson (1958) had already distinguished these under the names *Phyllocolpa piliserra*, *Phyllocolpa scotaspis* and *Phyllocolpa anglica*. Kopelke (2007b) stated that only two European species occur on this host, and synonymised *Nematus anglicus* and *Nematus nigrolineatus* with *Phyllocolpa scotaspis*, thus leaving the taxon formerly referred to as *Phyllocolpa anglica* without a valid name. The opinions of Benson and Vikberg are followed here.

#### Phyllocolpa carinifrons

(Benson, 1940)

Pontania carinifrons  Benson, 1940
Phyllocolpa excavata (Marlatt, 1896): misident.
Phyllocolpa destricta (MacGillivray, 1923): misident.
Phyllocolpa apicifrons (Malaise, 1931): misident.

##### Distribution

England, Scotland, Ireland

##### Notes

Taxonomy follows Kopelke (2007a).

#### Phyllocolpa erythropyga

(Förster, 1854)

Nematus erythropygus  Förster, 1854
Phyllocolpa leucosticta (Hartig, 1837): misident.

##### Distribution

Scotland

##### Notes

Added by Liston et al. (2012).

#### Phyllocolpa ischnocera

(Thomson, 1863)

Nematus ischnocerus  Thomson, 1863
Nematus leucostigma (Cameron, 1876, *Nematus*)

##### Distribution

Scotland

##### Notes

Taxonomy follows Kopelke (2007a).

#### Phyllocolpa leucapsis

(Tischbein, 1846)

Nematus leucapsis  Tischbein, 1846
Nematus coriacea (Benson, 1953, *Nematus*)

##### Distribution

England, Scotland, Wales, Ireland

##### Notes

According to Kopelke (2007c) *Phyllocolpa coriacea* is a synonym of *Phyllocolpa leucapsis* (as stated in the abstract and table 1, whereas the synonymy with *Phyllocolpa alienata* on p. 153 is wrong; J.-P. Kopelke, pers. comm.). The concept of *Phyllocolpa leucapsis* in Kopelke (2007c) is different to that of Benson (1958) and most other previous taxonomists.

#### Phyllocolpa leucosticta

(Hartig, 1837)

Nematus leucostictus  Hartig, 1837
Nematus sharpi (Cameron, 1876, *Nematus*)

##### Distribution

England, Scotland, Wales, Ireland

##### Notes

According to Kopelke (2007a), *Phyllocolpa erythropyga* (Förster, 1854) has previously been confused with *Phyllocolpa leucosticta*. Whilst *Phyllocolpa erythropyga* was recorded in the British Isles by Liston et al. (2012), records of *Phyllocolpa leucosticta* require validation.

#### Phyllocolpa oblita

(Serville, 1823)

Nematus oblitus  Serville, 1823
Nematus puella (Thomson, 1871, *Nematus*)

##### Distribution

England, Wales

#### Phyllocolpa piliserra

(Thomson, 1863)

Nematus piliserra  Thomson, 1863
Phyllocolpa xanthogaster (Förster, 1854): Cameron, 1877 misident.

##### Distribution

England, Scotland, Ireland

#### Phyllocolpa plicalapponum

Kopelke, 2007

##### Distribution

Scotland

##### Notes

Added by Liston et al. (2012).

#### Phyllocolpa plicaphylicifolia

Kopelke, 2007

##### Distribution

Scotland

##### Notes

Added by Liston et al. (2012).

#### Phyllocolpa polita

(Zaddach, 1883)

Nematus politus  Zaddach, 1883

##### Distribution

Scotland

##### Notes

Regarded as a valid species by Kopelke (2003), Kopelke (2007a). Material from Scotland was mentioned by Brischke (1884).

#### Phyllocolpa prussica

(Zaddach, 1883)

Nematus prussicus  Zaddach, 1883

##### Distribution

Scotland

##### Notes

Added by Liston et al. (2012).

#### Phyllocolpa scotaspis

(Förster, 1854)

Nematus scotaspis  Förster, 1854
Nematus westermanni (Boheman, 1852, *Nematus*) nom. ob.
Nematus westermanni (Thomson, 1863, *Nematus*)

##### Distribution

England, Scotland, Ireland

#### Pontania

Costa, 1852

EUPONTANIA Zinovjev, 1985
TUBPONTANIA Vikberg, 2010

##### Notes

Subsequent to the revision of the *Pontania viminalis* species group by Kopelke (1991), numerous important taxonomic and nomenclatural changes, followed here, were proposed by Vikberg (2003) and Vikberg and Zinovjev (2006).

#### Pontania anomaloptera

(Förster, 1854)

Nematus anomalopterus  Förster, 1854
Nematus tuberculata (Benson, 1953, *Nematus*)

##### Distribution

England, Scotland, Wales, Ireland

##### Notes

Taxonomy of the species follows Vikberg (2010b) (under *Tubpontania anomaloptera*).

#### Pontania aquilonis

Benson, 1941

Pontania algida Benson, 1941

##### Distribution

Scotland

#### Pontania arbusculae

Benson, 1941

##### Distribution

Scotland

#### Pontania arcticornis

Konow, 1904

Pontania phylicifoliae Forsius, 1920
Pontania hepatimaculae Malaise, 1920

##### Distribution

England, Scotland, Ireland

#### Pontania brevicornis

(Förster, 1854)

Nematus brevicornis  Förster, 1854
Nematus curticornis (Cameron, 1885, *Nematus*)
Pontania saliciscinereae (Retzius, 1783): misident.
Pontania kriechbaumeri Konow, 1901: misident.
Pontania pedunculi (Hartig, 1837): Kopelke 1991 misident.

##### Distribution

England, Scotland

#### Pontania bridgmanii

(Cameron, 1883)

Nematus bridgmanii  Cameron, 1883
Pontania capreae (Linnaeus, 1758): misident.

##### Distribution

England, Scotland, Wales, Ireland

#### Pontania collactanea

(Förster, 1854)

Nematus collactaneus  Förster, 1854
Nematus vacciniellus (Cameron, 1876, *Nematus*)

##### Distribution

England, Scotland, Ireland

#### Pontania crassipes

(Thomson, 1871)

Nematus crassipes  Thomson, 1871
Pontania lapponica Malaise, 1920

##### Distribution

Scotland

#### Pontania dolichura

(Thomson, 1871)

Nematus dolichurus  Thomson, 1871
Pontania lapponicola Kopelke, 1994

##### Distribution

Scotland

##### Notes

Added on the basis of records of galls of *Pontania dolichura* from *Salix lapponum* in Benson (1954). See Vikberg and Malinen (2012) on taxonomy.

#### Pontania femoralis

(Cameron, 1876)

Nematus femoralis  Cameron, 1876
Pontania robbinsi Benson, 1935
Pontania dolichura (Thomson, 1871): misident.

##### Distribution

England, Scotland

##### Notes

See Vikberg and Malinen (2012) on taxonomy.

#### Pontania herbaceae

(Cameron, 1876)

Nematus herbaceae  Cameron, 1876
Pontania crassipes (Thomson, 1871): misident.

##### Distribution

England, Scotland, Wales, Ireland

#### Pontania myrsiniticola

Kopelke, 1991

##### Distribution

Scotland

##### Notes

Added by Liston and Blank (2006).

#### Pontania nigricantis

Kopelke, 1986

Pontania dolichura (Thomson, 1871): misident.

##### Distribution

England, Scotland

##### Notes

Added on the basis of records of galls of *Pontania dolichura* from *Salix nigricans* in Benson (1954).

#### Pontania pedunculi

(Hartig, 1837)

Nematus pedunculi  Hartig, 1837
Nematus bella (Zaddach, 1876, *Nematus*)
Nematus baccarum (Cameron, 1876, *Nematus*)
Pontania pusilla Lindqvist, 1964
Pontania gallarum (Hartig, 1837): Kopelke, 1991 misident.

##### Distribution

England, Scotland, Ireland

#### Pontania proxima

(Serville, 1823)

Nematus proximus  Serville, 1823
Nematus gallicola (Stephens, 1835, *Nematus*)
Euura flavipes (Cameron, 1885, *Euura*)
Pontania capreae (Linnaeus, 1758): misident.

##### Distribution

England, Scotland, Wales, Ireland

#### Pontania purpureae

(Cameron, 1884)

Nematus purpureae  Cameron, 1884

##### Distribution

England

#### Pontania pustulator

Forsius, 1923

##### Distribution

England, Scotland

#### Pontania saliciscinereae

(Retzius, 1783)

Tenthredo saliciscinereae  Retzius, 1783
Nematus gallarum (Hartig, 1837, *Nematus*)
Nematus aestiva (Thomson, 1862, *Nematus*)
Pontania harrisoni Benson, 1940
Pontania varia Kopelke, 1991

##### Distribution

England, Scotland

##### Notes

Nomenclature follows Blank et al. (2009).

#### Pontania samolad

Malaise, 1920

##### Distribution

Scotland

#### Pontania triandrae

Benson, 1941

Pontania capreae (Linnaeus, 1758): misident.

##### Distribution

England, Scotland, Ireland

#### Pontania vesicator

(Bremi-Wolf, 1849)

Nematus vesicator  Bremi-Wolf, 1849
Nematus helicina (Brischke, 1850, *Nematus*)

##### Distribution

England, Scotland, Ireland

#### Pontania viminalis

(Linnaeus, 1758)

Cynips viminalis  Linnaeus, 1758
Nematus vollenhoveni (Cameron, 1874, *Nematus*)
Nematus ?interstitialis (Cameron, 1876, *Nematus*)
Pontania hungarica Enslin, 1918

##### Distribution

England, Scotland, Wales, Ireland

#### Pristiphorini

Vikberg, 1982

#### Pristicampus

Zinovjev, 1993

#### Pristicampus arcticus

(Lindqvist, 1958)

Mesoneura arctica  Lindqvist, 1959
Pachynematus arcticus : Benson, 1961

##### Distribution

Scotland

#### Pristiphora

Latreille, 1810

##### Notes

Species of *Pristiphora* removed from the British and Irish list:

[***Lygaeonematus karvoneni*** (Lindqvist, 1952, *Lygaeonematus*)] Recorded by Liston (1983a) but based on a misidentification.

[***Pristiphora micronematica*** Malaise, 1931] Recorded by Liston (1982) but based on a misidentification.

#### Lygaeonematus

Konow, 1890

#### Pristiphora (Lygaeonematus) abietina

(Christ, 1791)

Tenthredo abietina  Christ, 1791
Tenthredo pini (Retzius, 1783, *Tenthredo*) preocc.

##### Distribution

England, Scotland, Wales, Ireland

#### Pristiphora (Lygaeonematus) compressa

(Hartig, 1837)

Nematus compressus  Hartig, 1837

##### Distribution

England, Scotland

#### Pristiphora (Lygaeonematus) decipiens

(Enslin, 1916)

Lygaeonematus decipiens  Enslin, 1916

##### Distribution

England, Scotland

##### Notes

Added Liston (1981b).

#### Pristiphora (Lygaeonematus) erichsonii

(Hartig, 1837)

Nematus erichsonii  Hartig, 1837
Nematus leachii (Dahlbom, 1835, *Nematus*): suppressed

##### Distribution

England, Scotland, Wales, Ireland

#### Pristiphora (Lygaeonematus) glauca

Benson, 1954

Pachynematus ?laricivora (Takagi, 1931, *Pachynematus*) preocc.

##### Distribution

England, Wales

#### Pristiphora (Lygaeonematus) pseudodecipiens

Beneš & Kristek, 1976

Pristiphora decipiens (Enslin, 1916): misident.

##### Distribution

England, Scotland

##### Notes

Added by Beneš and Krístek (1976).

#### Pristiphora (Lygaeonematus) saxesenii

(Hartig, 1837)

Nematus saxesenii  Hartig, 1837
Pristiphora thalenhorsti Wong, 1975

##### Distribution

England, Scotland, Wales

#### Pristiphora (Lygaeonematus) subarctica

(Forsslund, 1936)

Lygaeonematus subarcticus  Forsslund 1936
Pristiphora pseudosaxesenii Lindqvist, 1968

##### Distribution

England

#### Pristiphora (Lygaeonematus) wesmaeli

(Tischbein, 1853)

Nematus wesmaeli  Tischbein, 1853

##### Distribution

England, Scotland, Wales, Ireland

#### Lygaeophora

Liston, 1993

LYGAEOPHORA Lindqvist, 1952 unavailable name

#### Pristiphora (Lygaeophora) sermola

Liston, 1993

Lygaeonematus variipes (Lindqvist, 1952, *Lygaeonematus*) preocc.
Pristiphora lanifica (Zaddach, 1883): Liston, 1981a misident.

##### Distribution

Scotland

##### Notes

Added by Liston (1981a).

*Lygaeophora variipes* Lindqvist, 1952 is preoccupied in *Pristiphora* by *Pristiphora varipes* Serville, 1823. The names *Pristiphora varipes* and *Pristiphora variipes* are to be regarded as identical spellings: Article 58.14. (ICZN 1999). *Pristiphora varipes* Serville, 1823 is a species inquirenda, not a synonym of Cladius (Priophorus) pallipes Serville, 1823 or *Cladius brullei* (Dahlbom, 1835) (see Lacourt 2000). The conditions of Article 23.9., permitting continued use of the junior homonym as valid, are not fulfilled for *Pristiphora variipes* (Lindqvist) because this has only been used as a valid name on twenty occasions in works published within the last 50 years (23.9.1.2.).

#### Lygaeotus

Liston, 1993

LYGAEOTUS Lindqvist, 1952 unavailable name

#### Pristiphora (Lygaeotus) albilabris

(Boheman, 1852)

Nematus albilabris  Boheman, 1852
Nematus albilabris (Thomson, 1863, *Nematus*) preocc.

##### Distribution

England

#### Pristiphora (Lygaeotus) borea

(Konow, 1904)

Lygaeonematus boreus  Konow, 1904
Nematus astuta (Cameron, 1885, *Nematus*) nom. ob.
Pachynematus lapponica (Enslin, 1916, *Pachynematus*)

##### Distribution

Scotland

##### Notes

Taxonomy follows Liston et al. (2006).

#### Pristiphora (Lygaeotus) breadalbanensis

(Cameron, 1882)

Nematus breadalbanensis  Cameron, 1882
Lygaeonematus tromsoensis (Kiaer, 1898, *Lygaeonematus*)
Lygaeonematus corpulentus (Konow, 1904, *Lygaeonematus*)
Lygaeonematus arcticola (Enslin, 1916, *Lygaeonematus*)

##### Distribution

England, Scotland, Wales, Ireland

#### Pristiphora (Lygaeotus) carinata

(Hartig, 1837)

Nematus carinatus  Hartig, 1837
Tenthredo pallipes (Fallén, 1808, *Tenthredo*) preocc.

##### Distribution

England, Scotland, Wales

#### Pristiphora (Lygaeotus) coactula

(Ruthe, 1859)

Nematus coactulus  Ruthe, 1859
Nematus alpina (Thomson, 1871, *Nematus*)
Lygaeonematus pachyvalvis (Konow, 1904, *Lygaeonematus*)

##### Distribution

Scotland

#### Pristiphora (Lygaeotus) groenblomi

(Lindqvist, 1952)

Lygaeonematus grönblomi  Lindqvist, 1952
Pristiphora gronblomi : misspelling

##### Distribution

Scotland

##### Notes

Predominant usage is of this spelling, not *Pristiphora gronblomi*, which would be correct according to ICZN Article 32.5.2 (ICZN 1999). Following ICZN (Article 33.3.1) the use of the predominant spelling is maintained.

#### Pristiphora (Lygaeotus) lativentris

(Thomson, 1871)

Nematus lativentris  Thomson, 1871
Nematus scotica (Cameron, 1881, *Nematus*)
Nematus extrema (Holmgren, 1883, *Nematus*)

##### Distribution

England, Scotland

#### Pristiphora (Lygaeotus) mollis

(Hartig, 1837)

Nematus mollis  Hartig, 1837
Nematus whitei (Cameron, 1878, *Nematus*)
Pachynematus kontkaneni (Lindqvist, 1960, *Pachynematus*)

##### Distribution

England, Scotland, Wales, Ireland

#### Pristiphora (Lygaeotus) pseudocoactula

(Lindqvist, 1952)

Lygaeonematus pseudocoactulus Lindqvist, 1952
Pristiphora pachyvalvis (Konow, 1904): Benson, 1934 misident.

##### Distribution

England, Scotland, Wales

#### Micronematus

Konow, 1890

GYMNONYCHUS Marlatt, 1896

#### Pristiphora (Micronematus) abbreviata

(Hartig, 1837)

Nematus abbreviatus  Hartig, 1837
Gymnonychus californica (Marlatt, 1896, *Gymnonychus*)

##### Distribution

England

#### Pristiphora (Micronematus) biscalis

(Förster, 1854)

Nematus biscalis  Förster, 1854

##### Distribution

England, Wales, Ireland

#### Pristiphora (Micronematus) maesta

(Zaddach, 1876)

Nematus maestus  Zaddach, 1876
Nematus brevicornis (Thomson, 1862, *Nematus*) preocc.
Nematus parvicornis (Kirby, 1882, *Nematus*)
Pachynematus insularis (Malaise, 1921, *Pachynematus*)
Pristiphora moesta : misspelling

##### Distribution

England

#### Pristiphora (Micronematus) monogyniae

(Hartig, 1840)

Nematus monogyniae  Hartig, 1840
Nematus hibernica (Cameron, 1878, *Nematus*)

##### Distribution

England, Wales, Ireland

#### Pristiphora (Micronematus) retusa

(Thomson, 1871)

Nematus retusus  Thomson, 1871

##### Distribution

Scotland

#### Oligonematus

Zhelochovtsev, 1988

#### Pristiphora (Oligonematus) friesei

(Konow, 1904)

Lygaeonematus friesei  Konow, 1904
Pristiphora funerula (Costa, 1859): misident.

##### Distribution

Scotland, Ireland

##### Notes

Added by O'Connor and Liston (1994).

#### Pristiphora (Oligonematus) laricis

(Hartig, 1837)

Nematus laricis  Hartig, 1837
Nematus leucocnemis (Förster, 1854, *Nematus*)
Nematus oblonga (Cameron, 1882, *Nematus*)
Nematus laricivora (Brischke, 1883, *Nematus*)
Pachynematus ravida (Konow, 1903, *Pachynematus*)

##### Distribution

England, Scotland, Wales, Ireland

#### Pristiphora

Latreille, 1810

DIPHADNUS Hartig, 1837

#### Pristiphora (Pristiphora) alpestris

(Konow, 1903)

Lygaeonematus alpestris Konow, 1903
Lygaeonematus strandi Konow, 1901: Benson, 1934 misident.

##### Distribution

England

#### Pristiphora (Pristiphora) aphantoneura

(Förster, 1854)

Nematus aphantoneurus  Förster, 1854
Tenthredo fulvipes (Fallén, 1808, *Tenthredo*) preocc.
Pristiphora vicina Serville, 1823: misident.

##### Distribution

England, Wales

##### Notes

See Vikberg (2006) on taxonomy.

#### Pristiphora (Pristiphora) appendiculata

(Hartig, 1837)

Nematus appendiculatus  Hartig, 1837
Pristiphora pallipes Serville, 1823, preocc.
Pristiphora grossulariae Walsh, 1866
Pristiphora rufipes Serville, 1823: misident.

##### Distribution

England, Scotland, Ireland

#### Pristiphora (Pristiphora) armata

(Thomson, 1862)

Nematus armatus  Thomson, 1862
Nematus crassicornis (Hartig, 1837, *Nematus*) preocc.
Nematus fletcheri (Cameron, 1884, *Nematus*)
Nematus nigricollis (Cameron, 1885, *Nematus*)

##### Distribution

England, Scotland, Wales, Ireland

#### Pristiphora (Pristiphora) bifida

(Hellén, 1948)

Nematus bifidus  Hellén, 1948

##### Distribution

Scotland

#### Pristiphora (Pristiphora) brevis

(Hartig, 1837)

Nematus brevis  Hartig, 1837
Nematus fumipennis (Thomson, 1871, *Nematus*)
Pristiphora fuscata Benson, 1943
Pristiphora thalictri (Kriechbaumer, 1884): misident.

##### Distribution

England, Ireland

#### Pristiphora (Pristiphora) cincta

Newman, 1837

Nematus quercus (Hartig, 1837, *Nematus*)

##### Distribution

England, Scotland, Wales, Ireland

##### Notes

Possibly two British species occur under this name: one mainly in the lowlands with hostplant *Betula*, the other montane with host *Vaccinium*. Whilst *Pristiphora cincta* seems to be the correct name for the more brightly coloured lowland taxon, until a revision is made of the numerous supposed synonyms of *Pristiphora cincta*, the name for the species on *Vaccinium* is unclear.

#### Pristiphora (Pristiphora) confusa

Lindqvist, 1955

##### Distribution

England, Scotland, Wales

#### Pristiphora (Pristiphora) coniceps

Lindqvist, 1955

##### Distribution

England, Scotland

#### Pristiphora (Pristiphora) conjugata

(Dahlbom, 1835)

Nematus conjugatus  Dahlbom, 1835

##### Distribution

England

#### Pristiphora (Pristiphora) denudata

Konow, 1902

Pristiphora atlantica Lacourt, 1987: misident.

##### Distribution

England, Scotland, Wales, Ireland

##### Notes

Contrary to the opinion of Lacourt (1987), we do not consider this taxon to be synonymous with *Pristiphora pallidiventris* (Fallén, 1808) (*Pristiphora pallidiventris pallidiventris* sensu Lacourt), nor with *Pristiphora atlantica* Malaise, 1939 (= *Pristiphora atlantica* Lacourt, 1987) as previously treated by Liston (1995), who replaced the name *Pristiphora denudata* with *Pristiphora atlantica* Lacourt on the grounds that the name *Pristiphora denudata* is preoccupied in *Pristiphora* by *Nematus denudatus* Hartig, 1840. *Nematus denudatus* was at that time considered to be a synonym of *Pristiphora carinata* (Hartig, 1837). According to Schmidt (in Lacourt 1999), *Nematus denudatus* Hartig actually belongs to Amauronematus (Brachycoluma), thus removing the homonymy. *Pristiphora denudata* differs from *Pristiphora pallidiventris* not only in the more extensively pale abdomen (without intermediate specimens), but also in details of the structure of the saw, and is probably attached only to *Rubus* species, whereas *Pristiphora pallidiventris* is polyphagous on various Rosaceae.

#### Pristiphora (Pristiphora) geniculata

(Hartig, 1840)

Nematus geniculatus  Hartig, 1840

##### Distribution

England, Ireland

#### Pristiphora (Pristiphora) insularis

Rohwer, 1910

Pristiphora kamtchatica Malaise, 1931
Pristiphora mesatlantica Lacourt, 1976
Pristiphora luteiventris Koch, 1989
Pristiphora paedida (Konow, 1904): misident.

##### Distribution

England, Scotland, Wales, Ireland

##### Notes

Synonymy follows Haris (2006).

#### Pristiphora (Pristiphora) leucopus

(Hellén, 1948)

Nematus leucopus  Hellén, 1948

##### Distribution

England, Scotland

##### Notes

Added by Grearson (2006).

#### Pristiphora (Pristiphora) luteipes

Lindqvist, 1955

Pristiphora pygmaea Lindqvist, 1964

##### Distribution

England, Scotland, Wales, Ireland

##### Notes

Added by O'Connor et al. (1997).

See Vikberg (2006) on taxonomy.

#### Pristiphora (Pristiphora) melanocarpa

(Hartig, 1840)

Nematus melanocarpus  Hartig, 1840
Nematus wuestneii (R. Stein, 1885, *Nematus*)

##### Distribution

England, Scotland, Wales, Ireland

#### Pristiphora (Pristiphora) pallidiventris

(Fallén, 1808)

Tenthredo pallidiventris  Fallén, 1808
Nematus ephippiger (Hartig, 1840, *Nematus*)
Nematus flavicoma (Tischbein, 1840, *Nematus*)
Nematus nigricans (Eversmann, 1847, *Nematus*)
Nematus caudalis (Eversmann, 1847, *Nematus*)
Nematus breviuscula (Eversmann, 1847, *Nematus*)
Nematus gemella (Förster, 1854, *Nematus*)
Nematus cirrhostoma (Zaddach, 1883, *Nematus*)
Pristiphora myosotidis (Fabricius, 1804): Stephens, 1835 misident.
Nematus obductus (Hartig, 1837): Cameron, 1874 misident.

##### Distribution

England, Scotland, Wales, Ireland

#### Pristiphora (Pristiphora) punctifrons

(Thomson, 1871)

Nematus punctifrons  Thomson, 1871

##### Distribution

England, Scotland, Wales, Ireland

#### Pristiphora (Pristiphora) ruficornis

(Olivier, 1811)

Nematus ruficornis  Olivier, 1811
Nematus fraxini (Hartig, 1837, *Nematus*)

##### Distribution

England, Scotland, Wales, Ireland

#### Pristiphora (Pristiphora) rufipes

Serville, 1823

Nematus aquilegiae (Vollenhoven, 1866, *Nematus*)
Pristiphora alnivora (Hartig, 1840): misident.

##### Distribution

England, Scotland

#### Pristiphora (Pristiphora) staudingeri

(Ruthe, 1859)

Nematus staudingeri  Ruthe, 1859
Pristiphora circularis Kincaid, 1900
Pristiphora ?hyperborea Malaise, 1921
Pristiphora asperlatus Benson, 1935

##### Distribution

England, Scotland, Wales, Ireland

##### Notes

*Pristiphora hyperborea* was recorded as British by Benson (1935), but later treated by him as a synonym of *Pristiphora staudingeri*. V. Vikberg considers *Pristiphora hyperborea* to represent a valid species (see Zhelochovtsev 1988). Probably both species occur in the British Isles.

#### Pristiphora (Pristiphora) subbifida

(Thomson, 1871)

Nematus subbifidus  Thomson, 1871

##### Distribution

England, Wales

##### Notes

Characterisation of *Pristiphora subbifida* by Zhelochovtsev (1988) is based on misidentification by Lindqvist (1973) of *Pristiphora conjugata*, or a similar species.

#### Pristiphora (Pristiphora) subopaca

Lindqvist, 1955

##### Distribution

England

##### Notes

Recorded by Lindqvist (1955) from Great Britain, but in the reprints of this paper which he distributed, “Grossbritannien” is scored out. Therefore this record is considered to be based on a misidentification. However, J. Grearson has recently taken females in England, identity confirmed by V. Vikberg after comparison with the holotype (Grearson, in preparation).

#### Pristiphora (Pristiphora) testacea

(Jurine, 1807)

Pteronus testaceus  Jurine, 1807
Tenthredo betulae (Retzius, 1783, *Tenthredo*) preocc.
Nematus crassiventris (Cameron, 1878, *Nematus*)

##### Distribution

England, Scotland, Wales, Ireland

#### Pristiphora (Pristiphora) thalictri

(Kriechbaumer, 1884)

Nematus thalictri  Kriechbaumer, 1884

##### Distribution

Scotland

##### Notes

Added by Liston et al. (2012).

#### Sharliphora

Wong, 1969

#### Pristiphora (Sharliphora) amphibola

(Förster, 1854)

Nematus amphibolus  Förster, 1854
Nematus laeta (Cameron, 1883, *Nematus*)
Nematus fraterna (Cameron, 1885, *Nematus*)

##### Distribution

England, Scotland, Wales

#### Pristiphora (Sharliphora) nigella

(Förster, 1854)

Nematus nigellus  Förster, 1854
Tenthredo ambigua (Fallén, 1808, *Tenthredo*) preocc.
Nematus furvescens (Cameron, 1876, *Nematus*)

##### Distribution

England, Scotland, Ireland

#### Pseudodineurini

Benson, 1938

#### Endophytus

Hering, 1934

NEOPELMATOPUS Conde, 1934

#### Endophytus anemones

(Hering, 1924)

Pelmatopus anemones  Hering, 1924
Pelmatopus tenuiserra (Lindqvist, 1949, *Pelmatopus*)

##### Distribution

England

#### Pseudodineura

Konow, 1885

PELMATOPUS Hartig, 1837 preocc.
PHYLLOPAIS Hering, 1934

#### Pseudodineura enslini

(Hering, 1923)

Pelmatopus enslini  Hering, 1923

##### Distribution

England, Scotland, Wales

#### Pseudodineura fuscula

(Klug, 1816)

Tenthredo fuscula  Klug, 1816
Dineura despecta (Hartig, 1837, *Dineura*)
Dineura simulans (Cameron, 1877, *Dineura*)

##### Distribution

England, Scotland, Ireland

#### Pseudodineura mentiens

(Thomson, 1871)

Blennocampa mentiens  Thomson, 1871

##### Distribution

Scotland

##### Notes

Added by Liston and Blank (2006).

#### Stauronematini

Lacourt, 1998

#### Stauronematus

Benson, 1953

STAURONEMA Benson, 1948 preocc.

#### Stauronematus platycerus

(Hartig, 1840)

Nematus platycerus  Hartig, 1840
Stauronematus compressicornis (Fabricius, 1804): misident.

##### Distribution

England, Scotland, Wales

#### Selandriinae

Thomson, 1871

#### Aneugmenini

Takeuchi, 1941

#### Aneugmenus

Hartig, 1837

ATOPOSELANDRIA Enslin, 1913

#### Aneugmenus coronatus

(Klug, 1818)

Tenthredo coronata  Klug, 1818
Selandria analis (Thomson, 1871, *Selandria*)
Selandria cereipes (Vollenhoven, 1873, *Selandria*)

##### Distribution

England, Scotland, Ireland

##### Notes

Not a synonym of *Aneugmenus padi*. See Chevin (1980). There is a major divergence in opinion regarding the association of the sexes of these species: compare, for example, Blank (1998), Chevin (1980) and Benson (1952), Benson (1968).

#### Aneugmenus fuerstenbergensis

(Konow, 1885)

Selandria fuerstenbergensis  Konow, 1885
Aneugmenus furstenbergensis : misspelling

##### Distribution

England, Scotland, Wales, Ireland

#### Aneugmenus padi

(Linnaeus, 1760)

Tenthredo padi  Linnaeus, 1760
Tenthredo stramineipes (Klug, 1816, *Tenthredo*)

##### Distribution

England, Scotland, Wales, Ireland

#### Aneugmenus temporalis

(Thomson, 1871)

Selandria temporalis  Thomson, 1871

##### Distribution

England, Scotland, Wales, Ireland

#### Dolerini

Thomson, 1871

#### Dolerus

Panzer, 1801

#### Achaetoprion

Goulet, 1986

JUNCILERUS Zhelochovtsev, 1988

#### Dolerus (Achaetoprion) ferrugatus

Serville, 1823

##### Distribution

England, Scotland, Wales, Ireland

#### Dolerus (Achaetoprion) madidus

(Klug, 1818)

Tenthredo madida  Klug, 1818
Tenthredo lateritius (Klug, 1818, *Tenthredo*)
Dolerus chappelli Cameron, 1877
Dolerus schulthessi Konow, 1887: ?misident.

##### Distribution

England, Scotland, Wales, Ireland

#### Dolerus (Achaetoprion) pachycerus

Hartig, 1837

Dolerus taeniatus Zaddach, 1859
Dolerus tinctipennis Cameron, 1881

##### Distribution

England, Scotland, Wales

#### Dolerus (Achaetoprion) triplicatus

(Klug, 1818)

Tenthredo triplicata  Klug, 1818
Dolerus steini Konow, 1885

##### Distribution

England, Wales

#### Cyperolerus

Zhelochovtsev, 1988

#### Dolerus (Cyperolerus) anticus

(Klug, 1818)

Tenthredo antica  Klug, 1818

##### Distribution

England

#### Dicrodolerus

Goulet, 1986

Tenthredo vestigialis  Klug, 1818

#### Dolerus (Dicrodolerus) vestigialis

(Klug, 1818)

Tenthredo vestigialis  Klug, 1818
Dolerus genucinctus Zaddach, 1859: Benson, 1934 misident.
Loderus vestigialis : Benson, 1952

##### Distribution

England, Scotland, Wales, Ireland

#### Dolerus

Panzer, 1801

DOLERUS Jurine, 1801 suppressed
DOSYTHEUS Leach, 1817

#### Dolerus (Dolerus) aericeps

Thomson, 1871

Dolerus bajulus Serville, 1823 nom. ob.

##### Distribution

England, Scotland, Wales, Ireland

#### Dolerus (Dolerus) bimaculatus

(Geoffroy, 1785)

Tenthredo bimaculata  Geoffroy, 1785
Tenthredo tristis (Fabricius, 1804, *Tenthredo*) preocc.

##### Distribution

England, Scotland, Wales, Ireland

#### Dolerus (Dolerus) cothurnatus

Serville, 1823

Tenthredo palustris (Klug, 1818, *Tenthredo*) preocc.
Dosytheus junci (Stephens, 1835, *Dosytheus*)

##### Distribution

England, Scotland, Wales, Ireland

#### Dolerus (Dolerus) germanicus

(Fabricius, 1775)

Tenthredo germanica  Fabricius, 1775
Dosytheus fuscipennis (Stephens, 1835, *Dosytheus*)
Dolerus fulviventris (Scopoli, 1763): Cameron, 1882 misident.
Dolerus pratensis (Linnaeus, 1758): misident.

##### Distribution

England, Scotland, Wales, Ireland

#### Dolerus (Dolerus) gessneri

André, 1880

Dolerus labiosus Konow, 1897

##### Distribution

England, Scotland, Wales, Ireland

#### Dolerus (Dolerus) yukonensis

Norton, 1872

Dolerus scoticus Cameron, 1881
Dolerus lateralis Konow, 1895
Dolerus arcticola Kiaer, 1898
Dolerus saxatilis Hartig, 1837: Benson, 1952 misident.

##### Distribution

England, Scotland, Wales

##### Notes

Haris (2000) regards *Dolerus scoticus* as a species distinct from *Dolerus yukonensis* (= *Dolerus arcticola* Kiaer). Benson (1935) had already drawn essentially the same conclusions. *Dolerus scoticus* was subsequently (Benson 1952) regarded as a synonym of *Dolerus saxatilis* and latterly (Benson 1958: corrigenda) as a synonym of *Dolerus yukonensis*. Goulet (1986) concurred with the synonymy of *Dolerus scoticus* and *Dolerus yukonensis*.

#### Equidolerus

Taeger & Blank, 1996

DOSYTHEUS : Goulet, 1986 misident.

#### Dolerus (Equidolerus) pratensis

(Linnaeus, 1758)

Tenthredo pratensis  Linnaeus, 1758
Tenthredo dubius (Klug, 1818, *Tenthredo*)
Tenthredo timidus (Klug, 1818, *Tenthredo*)

##### Distribution

England, Scotland, Wales, Ireland

#### Loderus

Konow, 1890

#### Dolerus (Loderus) gilvipes

(Klug, 1818)

Tenthredo gilvipes  Klug, 1818
Dolerus pratorum ssp. gilvipes (Klug, 1818)

##### Distribution

England, Scotland

#### Dolerus (Loderus) pratorum

(Fallén, 1808)

Tenthredo pratorum  Fallén, 1808

##### Distribution

England, Wales

#### Oncodolerus

Goulet, 1986

#### Dolerus (Oncodolerus) eversmanni

Kirby, 1882

Tenthredo palmatus (Klug, 1818, *Tenthredo*) preocc.
Loderus eversmanni : Benson, 1952

##### Distribution

England, Scotland, Wales, Ireland

#### Poodolerus

Zhelochovtsev, 1988

#### Dolerus (Poodolerus) aeneus

Hartig, 1837

Dolerus elongatus Thomson, 1871

##### Distribution

England, Scotland, Wales, Ireland

#### Dolerus (Poodolerus) anthracinus

(Klug, 1818)

Tenthredo anthracina  Klug, 1818

##### Distribution

England, Scotland, Wales

#### Dolerus (Poodolerus) asper

Zaddach, 1859

Dolerus carbonarius Zaddach, 1859
Dolerus oblongus Cameron, 1882
Dolerus planatus Hartig, 1837: misident.

##### Distribution

England, Scotland

##### Notes

Previous records of *Dolerus asper* from Wales and Ireland require checking, because they may refer to *Dolerus brevicornis* (below), recently distinguished by Heidemaa et al. (2004). English specimens of *Dolerus asper*, leg. K. J. Grearson, have been determined by M. Heidemaa. The only evidence for the presence of *Dolerus asper* in Scotland is the determination of a syntype specimen of *Dolerus oblongus* in NHM, examined by Heidemaa et al. (2004) and identified as *Dolerus asper*. Cameron (1882) described *Dolerus oblongus* from “Braemar, Rannoch, Clydesdale”.

#### Dolerus (Poodolerus) brevicornis

Zaddach, 1859

Dolerus asper Zaddach, 1859: misident.

##### Distribution

England, Scotland

##### Notes

Only recently distinguished from *Dolerus asper* (see above). A specimen collected by K. J. Grearson in England has been determined as *Dolerus brevicornis* by M. Heidemaa.

#### Dolerus (Poodolerus) coracinus

(Klug, 1818)

Tenthredo coracina  Klug, 1818

##### Distribution

England, Scotland

#### Dolerus (Poodolerus) fumosus

Stephens, 1835

Dolerus sanguinicollis (Klug, 1818): misident.

##### Distribution

England, Scotland, Wales

#### Dolerus (Poodolerus) gonager

(Fabricius, 1781)

Tenthredo gonagra  Fabricius, 1781

##### Distribution

England, Scotland, Wales

#### Dolerus (Poodolerus) haematodes

(Schrank, 1781)

Tenthredo haematodes  Schrank, 1781
Tenthredo collaris (Donovan, 1808, *Tenthredo*)

##### Distribution

England, Scotland, Wales, Ireland

#### Dolerus (Poodolerus) harwoodi

Benson, 1947

##### Distribution

Scotland

#### Dolerus (Poodolerus) liogaster

Thomson, 1871

##### Distribution

England, Scotland, Wales, Ireland

##### Notes

Heidemaa (2004) established that *Dolerus schmidti* (below) has hitherto been confused with *Dolerus liogaster*. *Dolerus liogaster* is confirmed as present in Britain by M. Heidemaa (pers. comm.) based on material collected by A. Barker.

#### Dolerus (Poodolerus) niger

(Linnaeus, 1767)

Tenthredo nigra  Linnaeus, 1767

##### Distribution

England, Scotland, Wales, Ireland

#### Dolerus (Poodolerus) nigratus

(Müller, 1776)

Tenthredo nigrata  Müller, 1776
Dolerus fissus Hartig, 1837

##### Distribution

England, Scotland, Wales, Ireland

#### Dolerus (Poodolerus) nitens

Zaddach, 1859

Dolerus coruscans Konow, 1890
Dolerus wanda Ross, 1935

##### Distribution

England, Scotland, Wales, Ireland

#### Dolerus (Poodolerus) picipes

(Klug, 1818)

Tenthredo picipes  Klug, 1818
Dolerus intermedius Cameron, 1881

##### Distribution

England, Scotland, Wales, Ireland

#### Dolerus (Poodolerus) possilensis

Cameron, 1882

Dolerus nitens Zaddach, 1859: misident.

##### Distribution

England, Scotland, Wales, Ireland

#### Dolerus (Poodolerus) puncticollis

Thomson, 1871

##### Distribution

England, Scotland, Wales, Ireland

#### Dolerus (Poodolerus) schmidti

Konow, 1884

##### Distribution

England, Scotland

##### Notes

See under *Dolerus liogaster* (above). *Dolerus schmidti* is present in Britain according to determinations made by M. Heidemaa: England, leg. K. J. Grearson and Scotland, leg. A. Barker.

#### Dolerus (Poodolerus) stygius

Förster, 1860

Dolerus megapterus Cameron, 1881

##### Distribution

England, Scotland, Wales

#### Dolerus (Poodolerus) varispinus

Hartig, 1837

Dolerus rugosus Konow, 1884
Dolerus rugosulus Dalla Torre, 1894
Dolerus brevitarsis : Benson, 1947 misident. and misspelling
Dolerus liogaster Thomson, 1871: misident.

##### Distribution

England, Scotland, Wales, Ireland

##### Notes

Taxonomy follows Heidemaa (2004).

#### Dulophanini

Lacourt, 1998

#### Birka

Malaise, 1944

#### Birka cinereipes

(Klug, 1816)

Tenthredo cinereipes  Klug, 1816
Tenthredo aperta (Hartig, 1837, *Tenthredo*)

##### Distribution

England, Scotland, Wales, Ireland

#### Nesoselandria

Rohwer, 1910

MELISANDRA Benson, 1939
DULOPHANES Konow, 1907: misident.

#### Nesoselandria morio

(Fabricius, 1781)

Tenthredo morio  Fabricius, 1781

##### Distribution

England, Scotland, Wales, Ireland

#### Selandriini

Thomson, 1871

#### Brachythops

Curtis, 1839

CORYNA Lepeletier & Serville, 1825 preocc.

#### Brachythops flavens

(Klug, 1816)

Tenthredo flavens  Klug, 1816
Tenthredo scapularis (Lepeletier & Serville, 1828, *Tenthredo*)
Brachythops seminigra Curtis, 1839
Selandria flavescens (Thomson, 1870, *Selandria*)

##### Distribution

England, Scotland, Wales, Ireland

#### Brachythops wuestneii

(Konow, 1885)

Selandria wuestneii  Konow 1885
Selandria flavistigma (Groenblom, 1939, *Selandria*)

##### Distribution

England, Scotland, Wales

#### Selandria

Leach, 1817

#### Selandria melanosterna

(Serville, 1823)

Tenthredo melanosterna  Serville, 1823
Selandria sixii Vollenhoven, 1858
Selandria grandis Zaddach, 1859
Selandria interstitialis Thomson, 1871
Selandria dorsalis Stephens, 1835: Kirby, 1882 misident.

##### Distribution

England, Scotland, Wales

#### Selandria serva

(Fabricius, 1793)

Tenthredo serva  Fabricius, 1793
Selandria dorsalis Stephens, 1835
Selandria excisa Konow, 1885
Selandria fuscitarsis Benson, 1954

##### Distribution

England, Scotland, Wales, Ireland

#### Strongylogastrini

Ashmead, 1898

#### Pseudohemitaxonus

Conde, 1932

#### Pseudohemitaxonus sharpi

(Cameron, 1879)

Strongylogaster sharpi  Cameron, 1879
Pseudohemitaxonus exsectus Conde, 1932
Pseudohemitaxonus mixtus (Klug, 1817): Cameron, 1874 misident.
Strongylogaster contigua (Konow, 1885): misident.

##### Distribution

England, Scotland

#### Stromboceros

Konow, 1885

STROMBOCERINA Malaise, 1942
STROMBOCERUS : misspelling

#### Stromboceros delicatulus

(Fallén, 1808)

Tenthredo delicatula  Fallén, 1808
Strongylogaster eborina (Klug, 1817, *Tenthredo*)
Selandria phthisica Vollenhoven, 1869

##### Distribution

England, Scotland, Wales, Ireland

#### Strongylogaster

Dahlbom, 1835

PSEUDOTAXONUS Costa, 1894

#### Strongylogaster filicis

(Klug, 1817)

Tenthredo filicis  Klug, 1817

##### Distribution

✝England

##### Notes

Extinct; recorded only on the basis of a single specimen by Bold (1873). The present whereabouts of the specimen are unknown.

#### Strongylogaster macula

(Klug, 1817)

Tenthredo macula  Klug, 1817
Thrinax intermedia (Konow, 1885, *Thrinax*)
Strongylogaster mixtus (Klug, 1817): Cameron, 1874 misident. and misspelling

##### Distribution

England, Scotland, Wales, Ireland

#### Strongylogaster mixta

(Klug, 1817)

Tenthredo mixta  Klug, 1817
Strongylogaster femoralis Cameron, 1875

##### Distribution

England, Scotland, Wales, Ireland

#### Strongylogaster multifasciata

(Geoffroy, 1785)

Tenthredo multifasciata  Geoffroy, 1785
Tenthredo lineata (Christ, 1791, *Tenthredo*)
Tenthredo atricornis (Stephens, 1835, *Tenthredo*)
Strongylogaster cingulata (Fabricius, 1793): misident.

##### Distribution

England, Scotland, Wales, Ireland

#### Strongylogaster xanthocera

(Stephens, 1835)

Tenthredo xanthocera  Stephens, 1829
Strongylogaster geniculata Thomson, 1871
Strongylogaster xanthoceros : misspelling

##### Distribution

England, Scotland, Wales

#### Tenthredininae

Latreille, 1802

#### Macrophyini

Benson, 1946

#### Macrophya

Dahlbom, 1835

PSEUDOMACROPHYA Enslin, 1913

#### Macrophya albicincta

(Schrank, 1776)

Tenthredo albicincta  Schrank, 1776

##### Distribution

England, Scotland, Wales

#### Macrophya albipuncta

(Fallén, 1808)

Tenthredo albipuncta  Fallén, 1808

##### Distribution

England, Scotland

#### Macrophya alboannulata

Costa, 1859

##### Distribution

England, Wales

##### Notes

Added by Liston (1983a).

#### Macrophya annulata

(Geoffroy, 1785)

Tenthredo annulata  Geoffroy, 1785
Tenthredo neglecta (Klug, 1817, *Tenthredo*)

##### Distribution

England, Wales

#### Macrophya blanda

(Fabricius, 1775)

Tenthredo blanda  Fabricius, 1775

##### Distribution

England

#### Macrophya duodecimpunctata

(Linnaeus, 1758)

Tenthredo duodecimpunctata  Linnaeus, 1758

##### Distribution

England, Scotland, Wales, Ireland

#### Macrophya montana

(Scopoli, 1763)

Tenthredo montana  Scopoli, 1763
Macrophya rustica (Linnaeus, 1758): misident.

##### Distribution

England, Scotland, Wales

#### Macrophya punctumalbum

(Linnaeus, 1767)

Tenthredo punctumalbum  Linnaeus, 1767
Tenthredo erythropus (Schrank, 1776, *Tenthredo*)
Macrophya parvula Konow, 1884: Liston, 1987 misident.

##### Distribution

England, Wales, Ireland

#### Macrophya ribis

(Schrank, 1781)

Tenthredo ribis  Schrank, 1781

##### Distribution

England, Wales

#### Macrophya rufipes

(Linnaeus, 1758)

Tenthredo rufipes  Linnaeus, 1758
Allantus ione (Newman, 1837, *Allantus*)
Macrophya haematopus (Panzer, 1801): Cameron, 1882 misident.

##### Distribution

England, Wales

#### Pachyprotasis

Hartig, 1837

#### Pachyprotasis antennata

(Klug, 1817)

Tenthredo antennata  Klug, 1817

##### Distribution

England, Scotland, Wales, Ireland

#### Pachyprotasis nigronotata

Kriechbaumer, 1874

##### Distribution

Wales

#### Pachyprotasis rapae

(Linnaeus, 1767)

Tenthredo rapae  Linnaeus, 1767

##### Distribution

England, Scotland, Wales, Ireland

#### Pachyprotasis simulans

(Klug, 1817)

Tenthredo simulans  Klug, 1817

##### Distribution

England, Scotland, Wales

#### Pachyprotasis variegata

(Fallén, 1808)

Tenthredo variegata  Fallén, 1808

##### Distribution

England, Scotland, Wales

#### Perineurini

Rohwer, 1911

#### Aglaostigma

W. F. Kirby, 1882

#### Aglaostigma aucupariae

(Klug, 1817)

Tenthredo aucupariae  Klug, 1817
Tenthredo solitaria (Fallén, 1808, *Tenthredo*) preocc.
Tenthredo juvenilis (Serville, 1823, *Tenthredo*)
Tenthredo gibbosa Fallén, 1808: Thomson, 1870 misident.

##### Distribution

England, Scotland, Wales, Ireland

#### Aglaostigma fulvipes

(Scopoli, 1763)

Tenthredo fulvipes  Scopoli, 1763
Tenthredo lateralis (Fabricius, 1779, *Tenthredo*)

##### Distribution

England, Scotland, Wales, Ireland

#### Perineura

Hartig, 1837

SYNAIREMA Hartig, 1837

#### Perineura rubi

(Panzer, 1803)

Allantus rubi  Panzer, 1803

##### Distribution

England, Scotland, Wales, Ireland

#### Tenthredopsis

Costa, 1859

EBOLIA Costa, 1859
THOMSONIA Konow, 1884
EUTENTHREDOPSIS Enslin, 1913

#### Tenthredopsis coquebertii

(Klug, 1817)

Tenthredo coquebertii  Klug, 1817
Tenthredopsis nigricollis Cameron, 1881
Tenthredopsis opulenta Konow, 1887
Tenthredopsis ignobilis (Klug, 1817): Cameron, 1882 misident.

##### Distribution

England, Scotland, Wales, Ireland

#### Tenthredopsis friesei

(Konow, 1884)

Thomsonia friesei  Konow, 1884
Tenthredo ?palmata  (Geoffroy, 1785, *Tenthredo*)
Thomsonia laticeps (Konow, 1884, *Thomsonia*)
Tenthredopsis korlevici Konow, 1887
Tenthredopsis arrogans Konow, 1890
Tenthredopsis pavida (Fabricius, 1775): misident.

##### Distribution

England, Scotland

#### Tenthredopsis litterata

(Geoffroy, 1785)

Tenthredo litterata  Geoffroy, 1785
Tenthredo carbonaria (Linnaeus, 1767, *Tenthredo*) preocc.
Tenthredo cordata (Geoffroy, 1785, *Tenthredo*)
Tenthredo microcephala (Serville, 1823, *Tenthredo*)
Tenthredo caliginosa (Stephens, 1835, *Tenthredo*)
Tenthredo femoralis (Stephens, 1835, *Tenthredo*)
Tenthredopsis nigriceps Cameron, 1881
Tenthredopsis nigronotata Cameron, 1881
Tenthredopsis pallida Konow, 1896

##### Distribution

England, Scotland, Wales

#### Tenthredopsis nassata

(Linnaeus, 1767)

Tenthredo nassata  Linnaeus, 1767
Tenthredo alneti (Schrank, 1781, *Tenthredo*)
Tenthredo dorsalis (Serville, 1823, *Tenthredo*)
Tenthredo fulviceps (Stephens, 1835, *Tenthredo*)
Tenthredo tristis (Stephens, 1835, *Tenthredo*)
Tenthredopsis albomaculata Cameron, 1881
Tenthredopsis dorsivittata Cameron, 1881
Tenthredopsis inornata Cameron, 1881
Tenthredopsis lividiventris Cameron, 1881
Tenthredopsis saundersi Cameron, 1881
Thomsonia elegans (Konow, 1884, *Thomsonia*)
Tenthredopsis gibberosa Konow, 1887
Tenthredopsis fenestrata Konow, 1890
Tenthredopsis tristior Morice, 1914
Tenthredopsis sordida (Klug, 1817): misident.

##### Distribution

England, Scotland, Wales, Ireland

#### Tenthredopsis ornata

(Serville, 1823)

Tenthredo ornata  Serville, 1823
Perineura excisa (Thomson, 1870, *Perineura*)
Tenthredopsis tesselata : Benson, 1968 misident. and misspelling

##### Distribution

England, Scotland

#### Tenthredopsis scutellaris

(Fabricius, 1804)

Tenthredo scutellaris  Fabricius, 1804
Tenthredo spreta (Serville, 1823, *Tenthredo*)
Tenthredopsis picticeps Cameron, 1881
Tenthredopsis flavomaculata Cameron, 1881
Tenthredopsis austriaca Konow, 1890
Tenthredopsis dubia Konow, 1890
Tenthredopsis parvula Konow, 1890
Tenthredopsis puncticollis Konow, 1890
Tenthredopsis thornleyi Konow, 1899
Tenthredopsis nassata : misident.
Tenthredopsis campestris (Linnaeus, 1758): misident.

##### Distribution

England, Scotland, Ireland

#### Tenthredinini

Latreille, 1802

SCIAPTERYGINI Benson, 1946

#### Cytisogaster

Lacourt, 1996

#### Cytisogaster chambersi

(Benson, 1947)

Rhogogaster chambersi  Benson, 1947

##### Distribution

England, Scotland, Wales

#### Cytisogaster genistae

(Benson, 1947)

Rhogogaster genistae  Benson, 1947

##### Distribution

England, Scotland, Wales

#### Cytisogaster picta

(Klug, 1817)

Tenthredo picta  Klug, 1817

##### Distribution

England, Wales

#### Rhogogaster

Konow, 1884

RHOGOGASTERA Konow, 1885

#### Rhogogaster chlorosoma

(Benson, 1943)

Tenthredo chlorosoma  Benson, 1943

##### Distribution

England, Scotland, Wales

#### Rhogogaster dryas

(Benson, 1943)

Tenthredo dryas  Benson, 1943
Rhogogaster similis Lindqvist, 1959

##### Distribution

England, Scotland

#### Rhogogaster punctulata

(Klug, 1817)

Tenthredo punctulata  Klug, 1817

##### Distribution

England, Scotland, Wales

#### Rhogogaster viridis

(Linnaeus, 1758)

Tenthredo viridis  Linnaeus, 1758
Tenthredo scalaris (Klug, 1817, *Tenthredo*)

##### Distribution

England, Scotland, Wales, Ireland

#### Sciapteryx

Stephens, 1835

SCIOPTERYX Cameron, 1882: misspelling

#### Sciapteryx consobrina

(Klug, 1816)

Tenthredo consobrina  Klug, 1816

##### Distribution

England

#### Sciapteryx soror

Konow, 1890

Sciapteryx costalis (Fabricius, 1775): misident.

##### Distribution

England, Scotland

#### Tenthredo

Linnaeus, 1758

TENTHREDELLA Rohwer, 1910
ELINORA Benson, 1940
EUROGASTER Zirngiebl, 1953
CUNEALA Zirngiebl, 1956
CEPHALEDO Zhelochovtsev, 1988
MACULEDO Zhelochovtsev, 1988
MURCIANA Lacourt, 1988
OLIVACEDO Zhelochovtsev, 1988
TEMULEDO Zhelochovtsev, 1988
ZONULEDO Zhelochovtsev, 1988
BLANKIA Lacourt, 1998

#### Tenthredo amoena

Gravenhorst, 1807

Tenthredo cingulum Klug, 1817
Allantus inversa (Costa, 1894, *Allantus*)

##### Distribution

England, Wales

#### Tenthredo arcuata

Forster, 1771

##### Distribution

England, Scotland, Wales, Ireland

#### Tenthredo atra

Linnaeus, 1758

Tenthredo dispar Klug, 1817
Tenthredo scotica Cameron, 1882

##### Distribution

England, Scotland, Wales, Ireland

#### Tenthredo baetica

Spinola, 1846

Tenthredo flavipes Geoffroy, 1785: Cameron, 1882 misident.
Tenthredo flaveola Gmelin, 1790: misident.

##### Distribution

England

##### Notes

The subspecies *Allantus baetica dominiquei* (Konow, 1894, *Allantus*) is represented in Britain; this taxon was placed for a time by Benson as a subspecies of the more continental *Tenthredo flaveola* Gmelin, 1790, but his opinion is not supported by data on distribution and morphology. Lacourt (1999) treated *Tenthredo dominiquei* as a northern subspecies of *Tenthredo baetica* (nominate subspecies confined to Spain and Portugal). The latter treatment is supported by results of COI barcoding (unpublished).

#### Tenthredo balteata

Klug, 1817

##### Distribution

England, Scotland, Wales, Ireland

#### Tenthredo brevicornis

(Konow, 1886)

Allantus brevicornis  Konow, 1886
Allantus nitidior (Konow, 1888, *Allantus*)
Tenthredo aegra Enslin, 1912
Tenthredo acerrima Benson, 1952
Tenthredo sulphuripes (Kriechbaumer, 1869): Benson, 1940 misident.

##### Distribution

England, Scotland, Wales, Ireland

#### Tenthredo colon

Klug, 1817

Tenthredo nigricollis Kirby, 1882

##### Distribution

England, Scotland, Wales, Ireland

#### Tenthredo distinguenda

(R. Stein, 1885)

Allantus distinguendus R. Stein, 1885

##### Distribution

England

#### Tenthredo fagi

Panzer, 1798

Tenthredo solitaria Scopoli, 1763: Cameron, 1882 misident.

##### Distribution

England, Scotland

#### Tenthredo ferruginea

Schrank, 1776

Tenthredo rufiventris Panzer, 1799

##### Distribution

England, Scotland, Wales, Ireland

#### Tenthredo ignobilis

Klug, 1817

##### Distribution

Scotland

##### Notes

Added by Liston et al. (2012).

#### Tenthredo livida

Linnaeus, 1758

##### Distribution

England, Scotland, Wales, Ireland

#### Tenthredo maculata

Geoffroy, 1785

##### Distribution

England, Scotland, Wales

#### Tenthredo mandibularis

Fabricius, 1804

##### Distribution

England, Scotland, Wales

#### Tenthredo mesomela

Linnaeus, 1758

Tenthredo mesomelas : misspelling

##### Distribution

England, Scotland, Wales, Ireland

#### Tenthredo mioceras

(Enslin, 1912)

Tenthredella mioceras  Enslin, 1912

##### Distribution

England, Scotland, Ireland

#### Tenthredo moniliata

Klug, 1817

Tenthredo lachlaniana Cameron, 1878

##### Distribution

England, Scotland, Wales, Ireland

#### Tenthredo neobesa

Zombori, 1980

Tenthredo pseudorossii Taeger, 1985
Tenthredo rossii (Panzer, 1803): Benson, 1952 misident.
Tenthredo temula Scopoli, 1763: Cameron misident.
Tenthredo vidua Rossi, 1790: Cameron misident.

##### Distribution

England

##### Notes

Possibly extinct in Britain.

#### Tenthredo notha

Klug, 1817

Allantus perkinsi (Morice, 1919, *Allantus*)
Tenthredo schaefferi Klug, 1817: Benson, 1959 misident.

##### Distribution

England, Scotland, Wales, Ireland

#### Tenthredo obsoleta

Klug, 1817

##### Distribution

England, Scotland, Ireland

#### Tenthredo olivacea

Klug, 1817

##### Distribution

England, Scotland, Wales

#### Tenthredo omissa

(Förster, 1844)

Allantus omissus  Förster, 1844
Tenthredo marginella Fabricius, 1793: Thomson, 1871 misident.

##### Distribution

England

#### Tenthredo schaefferi

Klug, 1817

##### Distribution

England

#### Tenthredo scrophulariae

Linnaeus, 1758

##### Distribution

England, Scotland, Wales

#### Tenthredo semicolon

Mol, 2013

##### Distribution

England

##### Notes

Added by Knight & Liston (in prep.).

#### Tenthredo temula

Scopoli, 1763

Tenthredo bicincta Linnaeus, 1767
Tenthredo celtica Benson, 1953

##### Distribution

England, Scotland, Wales, Ireland

#### Tenthredo thompsoni

(Curtis, 1839)

Allantus thompsoni  Curtis, 1839
Tenthredo marginella Fabricius, 1793: misident.

##### Distribution

England, Wales

#### Tenthredo velox

Fabricius, 1798

Tenthredo velox ab. nigrolineata Cameron, 1878 infrasubspecific name

##### Distribution

England, Scotland, Wales

#### Tenthredo vespa

Retzius, 1783

Tenthredo multifasciata Geoffroy, 1785: misident.
Tenthredo tricincta Fabricius, 1804
Tenthredo quadricincta Fallén, 1808

##### Distribution

England, Scotland, Wales

#### Tenthredo zona

Klug, 1817

Tenthredo quadricincta Fallén, 1808: Cameron, 1882 misident.

##### Distribution

England

### Superfamily XIPHYDRIOIDEA Leach, 1819

#### Xiphydriidae

Leach, 1819

#### Xiphydriinae

Leach, 1819

#### Xiphydria

Latreille, 1802

HYBONOTUS Klug, 1803
XIPHIURA Fallén, 1813
XIPHIDRIA Lamarck, 1817
HYPONOTUS Billberg, 1820
XYPHIDRIA Lepeletier, 1823
KONOWIA Brauns, 1884
PSEUDOXIPHYDRIA Enslin, 1911

#### Xiphydria camelus

(Linnaeus, 1758)

Ichneumon camelus  Linnaeus, 1758

##### Distribution

England, Scotland, Wales

#### Xiphydria longicollis

(Geoffroy, 1785)

Tenthredo longicollis  Geoffroy, 1785

##### Distribution

England

##### Notes

Added by Shaw and Liston (1985).

#### Xiphydria prolongata

(Geoffroy, 1785)

Tenthredo prolongata  Geoffroy, 1785
Sirex dromedarius Fabricius, 1787

##### Distribution

England, Wales

### Superfamily XYELOIDEA Newman, 1834

#### Xyelidae

Newman, 1834

#### Xyelinae

Newman, 1834

#### Xyelini

Newman, 1834

#### Xyela

Dalman, 1819

PINICOLA Brébisson, 1818 preocc.
XYELATANA Benson, 1938

#### Xyela curva

Benson, 1938

##### Distribution

England, Wales

##### Notes

Added by Liston and Blank (2006).

#### Xyela julii

(Brébisson, 1818)

Pinicola julii  Brébisson, 1818

##### Distribution

England, Scotland, Wales, Ireland

#### Xyela longula

Dalman, 1819

Xyela piliserra Thomson, 1871

##### Distribution

Scotland

## Figures and Tables

**Figure 1a.**
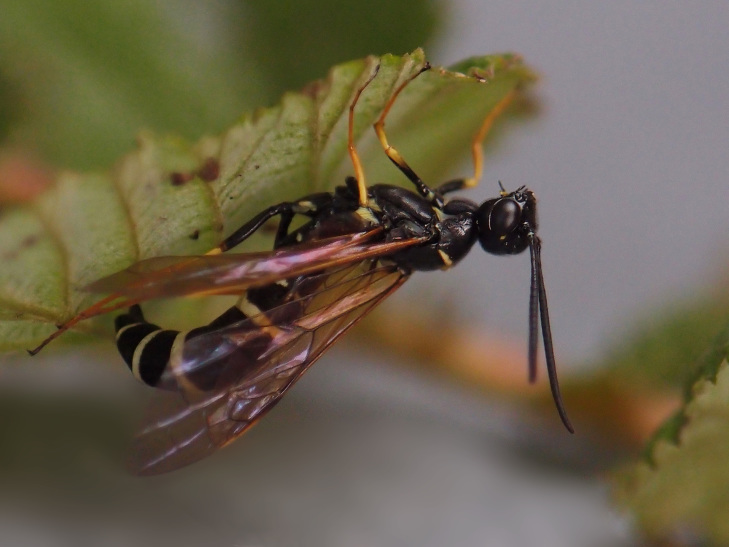
Cephidae, *Phylloecus xanthostoma* (G. Knight)

**Figure 1b.**
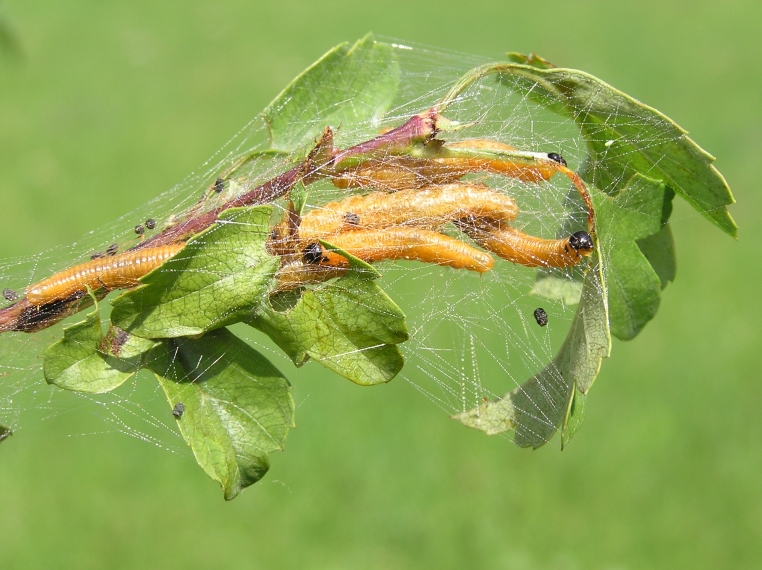
Pamphiliidae, *Neurotoma saltuum* larvae (G. Nowotny)

**Figure 1c.**
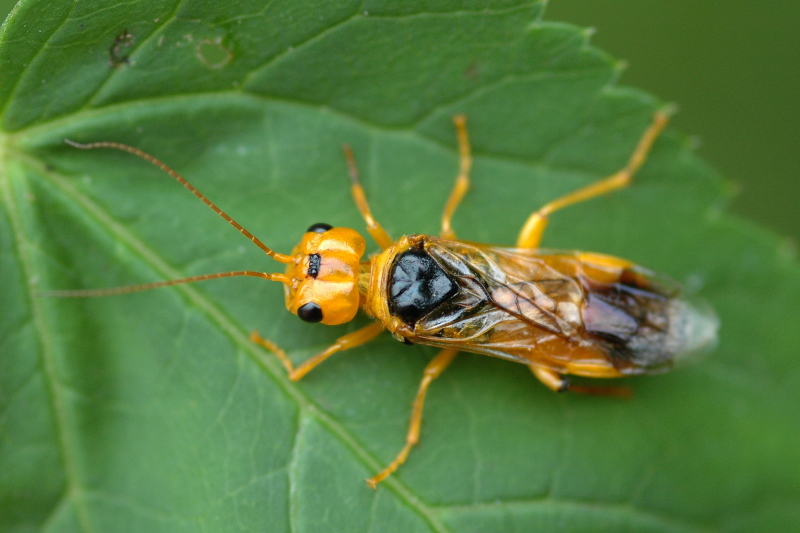
Pamphiliidae, *Pamphilius betulae* (H. Lewerenz)

**Figure 1d.**
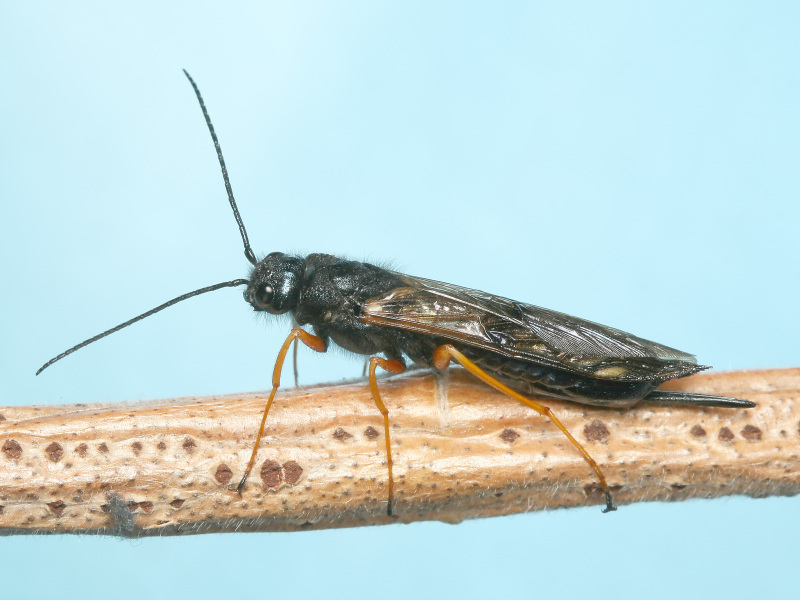
Siricidae, *Sirex noctilio* (H. Goulet)

**Figure 2a.**
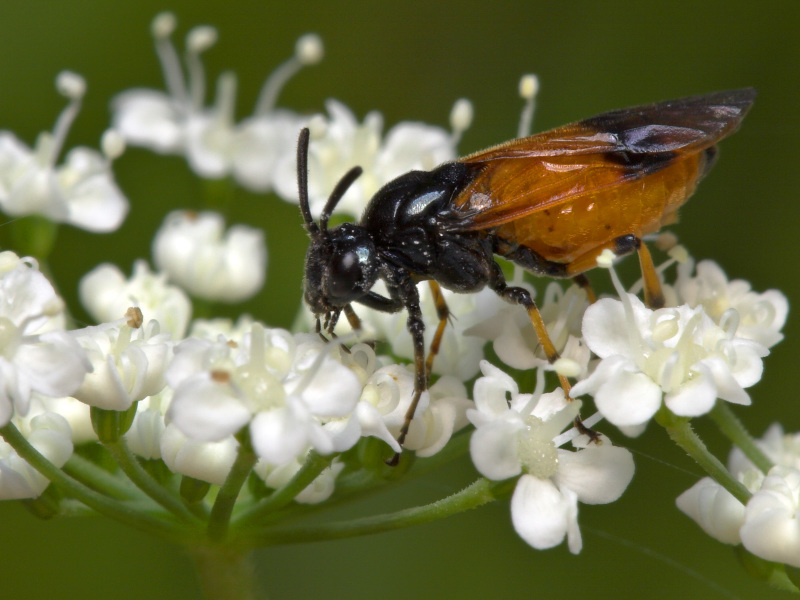
Argidae, *Arge cyanocrocea* (T. Tarvainis)

**Figure 2b.**
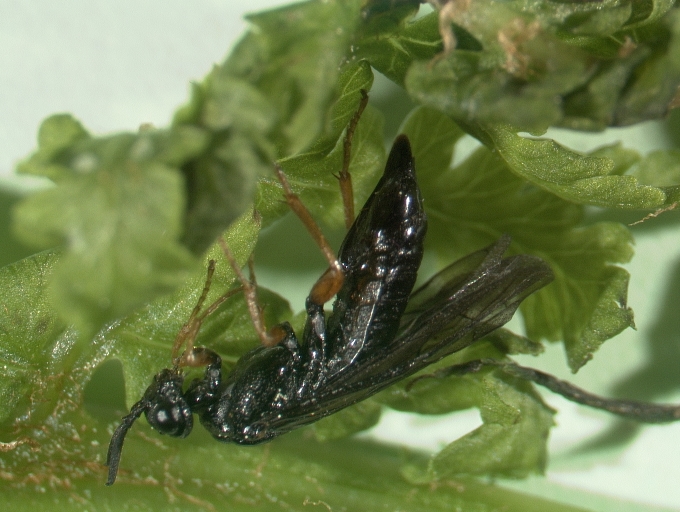
Blasticotomidae, *Blasticotoma filiceti* (A. Liston)

**Figure 2c.**
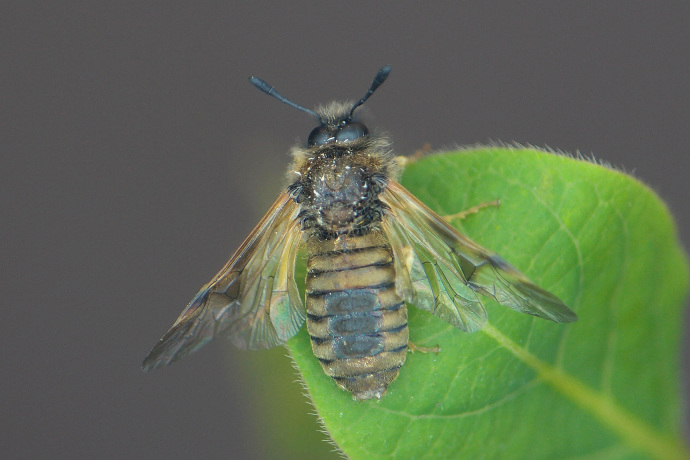
Cimbicidae, *Abia lonicerae* (G. Knight)

**Figure 2d.**
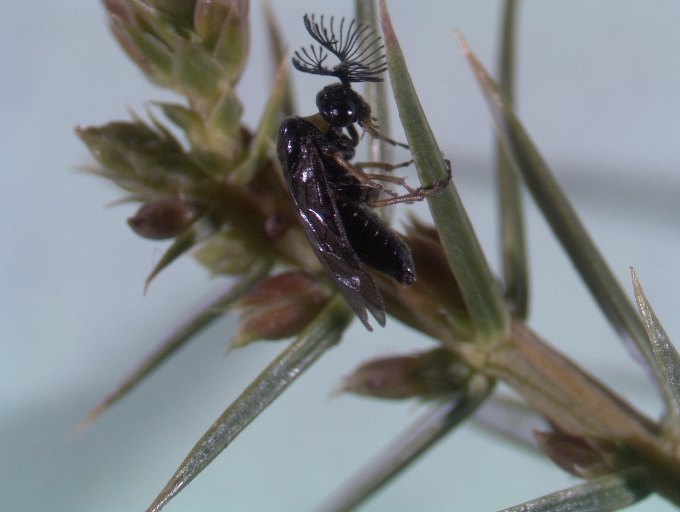
Diprionidae, *Monoctenus juniperi* (A. Taeger)

**Figure 3a.**
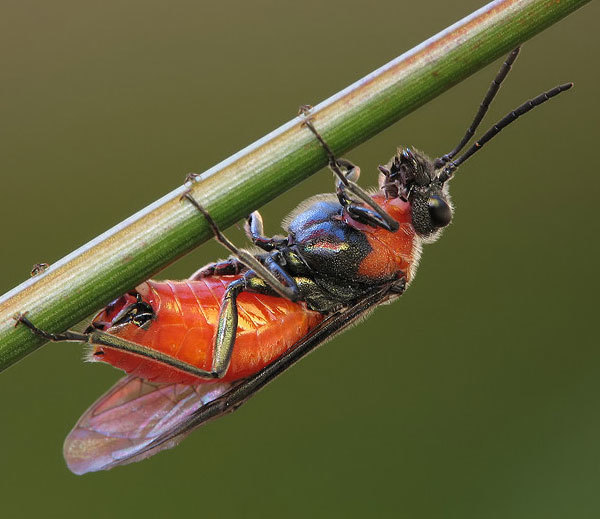
Tenthredinidae, *Dolerus madidus* (T. Thieme)

**Figure 3b.**
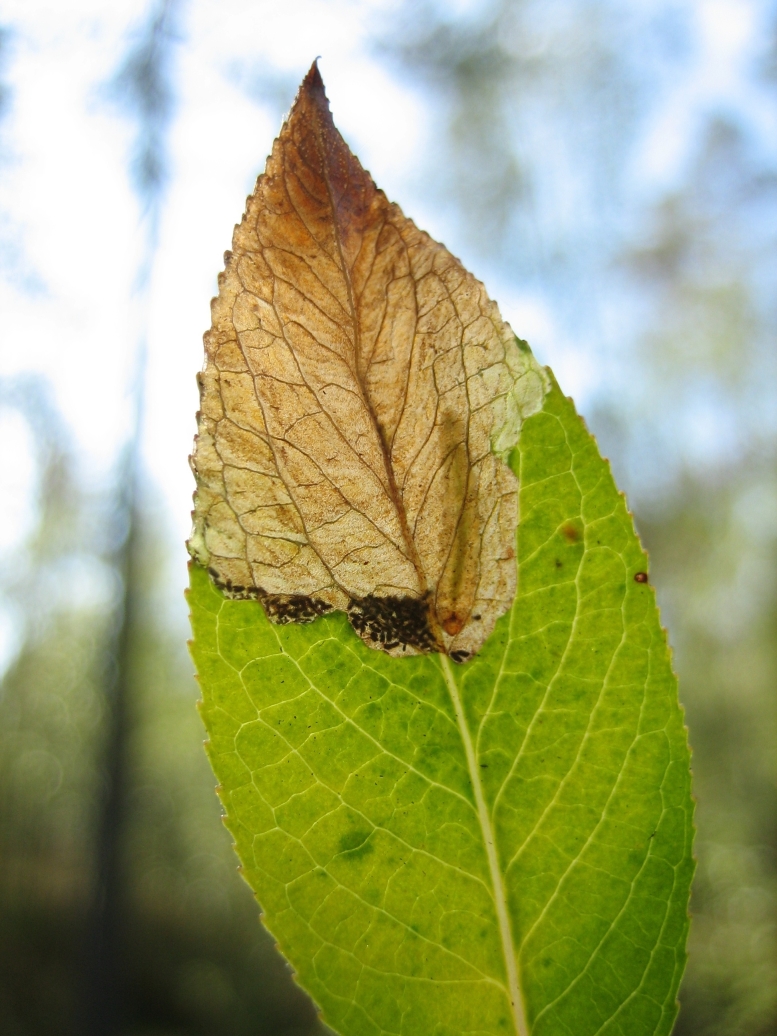
Tenthredinidae, *Heterarthrus microcephalus* larval mine (T. Nyman)

**Figure 3c.**
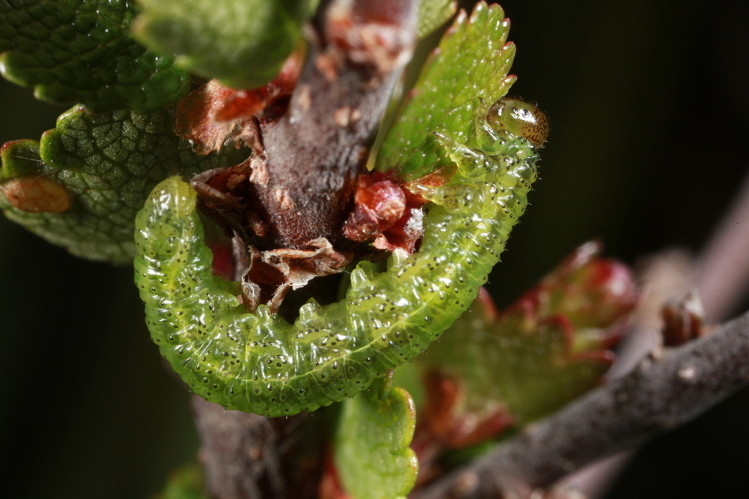
Tenthredinidae, *Amauronematus tristis* larva (A. Watson-Featherstone)

**Figure 3d.**
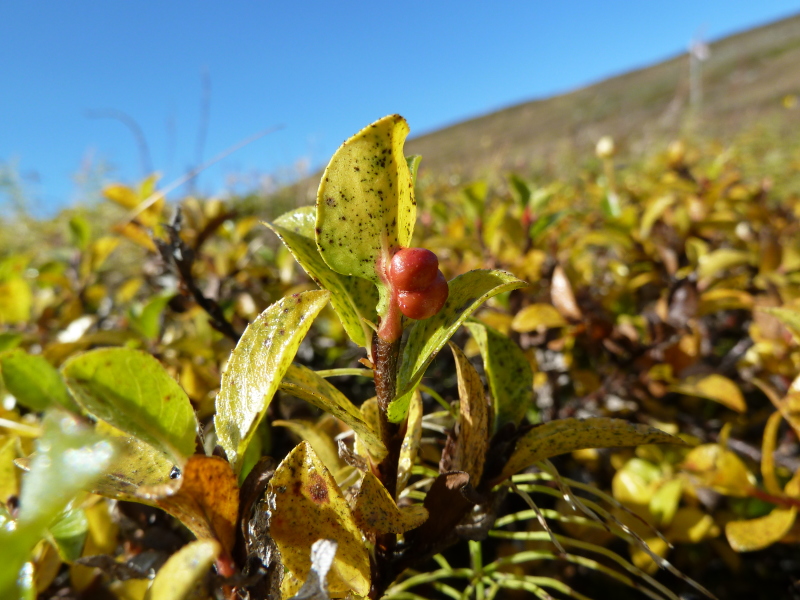
Tenthredinidae, *Pontania myrsiniticola* gall (A. Liston)

**Figure 4a.**
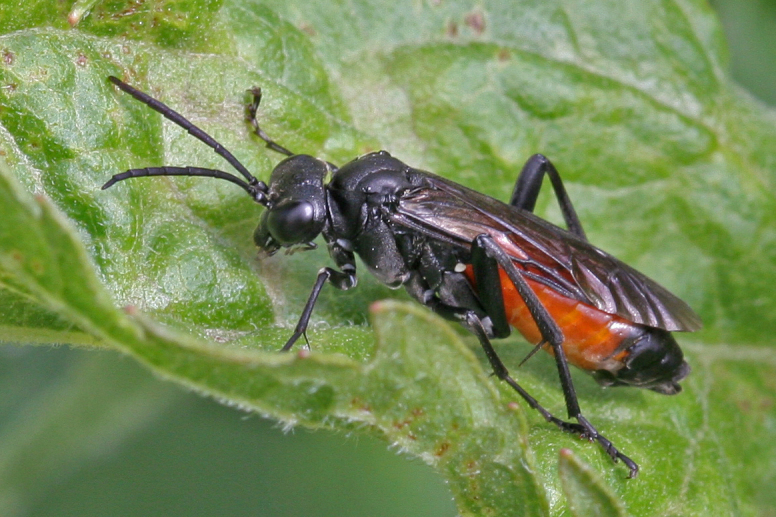
Tenthredinidae, *Macrophya annulata* (U. Rindlisbacher)

**Figure 4b.**
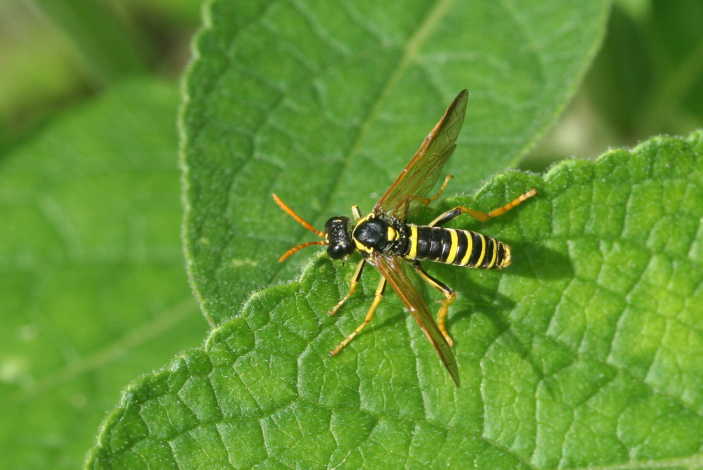
Tenthredinidae, *Tenthredo scrophulariae* (H. Savina)

**Figure 4c.**
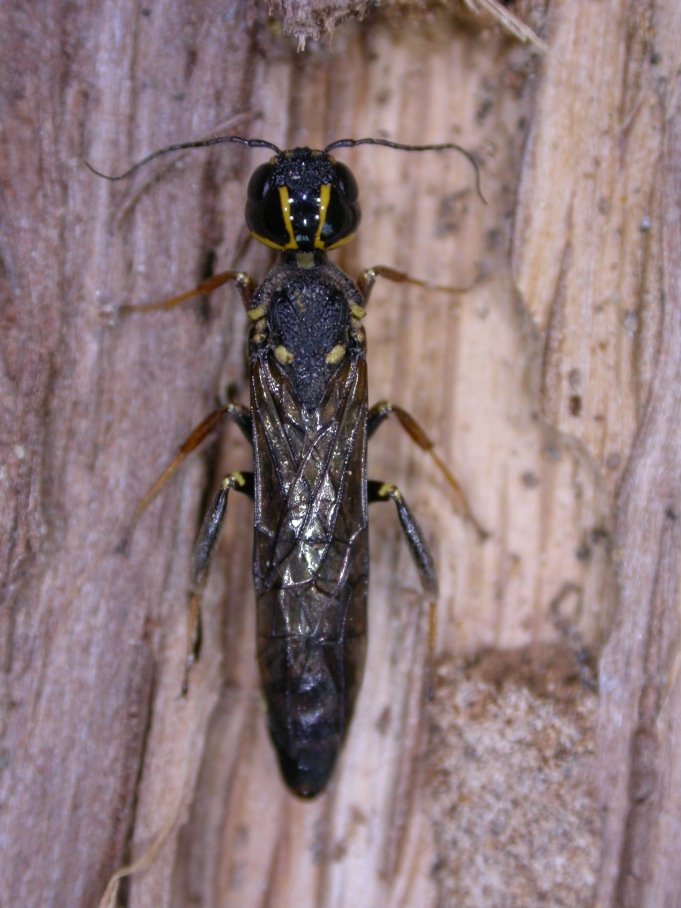
Xiphydriidae, *Xiphydria longicollis* (F. Stergulc)

**Figure 4d.**
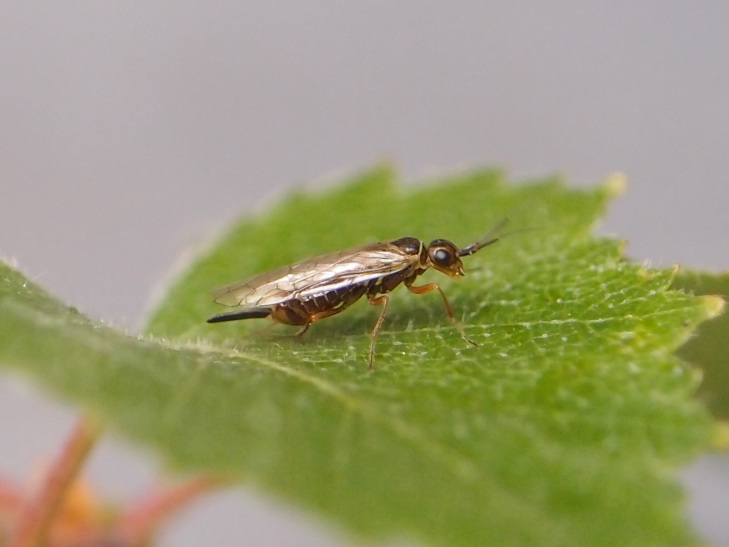
Xyelidae, *Xyela curva* (G. Knight)
